# Exploring how space, time, and sampling impact our ability to measure genetic structure across *Plasmodium falciparum* populations

**DOI:** 10.3389/fepid.2023.1058871

**Published:** 2023-02-17

**Authors:** Rohan Arambepola, Sophie Bérubé, Betsy Freedman, Steve M. Taylor, Wendy Prudhomme O’Meara, Andrew A. Obala, Amy Wesolowski

**Affiliations:** ^1^ Department of Epidemiology, Bloomberg School of Public Health, Johns Hopkins University, Batlimore, MD, United States; ^2^ Division of Infectious Diseases, Duke University Medical Center, Durham, NC, United States; ^3^ Duke Global Health Institute, Durham, NC, United States; ^4^ College of Health Sciences, Moi University, Eldoret, Kenya

**Keywords:** malaria, Plasmodium falciparum, simulation, relatedness, study design, agent - based modeling

## Abstract

A primary use of malaria parasite genomics is identifying highly related infections to quantify epidemiological, spatial, or temporal factors associated with patterns of transmission. For example, spatial clustering of highly related parasites can indicate foci of transmission and temporal differences in relatedness can serve as evidence for changes in transmission over time. However, for infections in settings of moderate to high endemicity, understanding patterns of relatedness is compromised by complex infections, overall high forces of infection, and a highly diverse parasite population. It is not clear how much these factors limit the utility of using genomic data to better understand transmission in these settings. In particular, further investigation is required to determine which patterns of relatedness we expect to see with high quality, densely sampled genomic data in a high transmission setting and how these observations change under different study designs, missingness, and biases in sample collection. Here we investigate two identity-by-state measures of relatedness and apply them to amplicon deep sequencing data collected as part of a longitudinal cohort in Western Kenya that has previously been analysed to identify individual-factors associated with sharing parasites with infected mosquitoes. With these data we use permutation tests, to evaluate several hypotheses about spatiotemporal patterns of relatedness compared to a null distribution. We observe evidence of temporal structure, but not of fine-scale spatial structure in the cohort data. To explore factors associated with the lack of spatial structure in these data, we construct a series of simplified simulation scenarios using an agent based model calibrated to entomological, epidemiological and genomic data from this cohort study to investigate whether the lack of spatial structure observed in the cohort could be due to inherent power limitations of this analytical method. We further investigate how our hypothesis testing behaves under different sampling schemes, levels of completely random and systematic missingness, and different transmission intensities.

## Introduction

*Plasmodium falciparum* genetic data can complement existing epidemiological surveillance strategies to inform transmission dynamics and patterns. For example, malaria genomics has been used to understand changes in transmission intensity (1) including following the implementation of interventions (2–4), to monitor the emergence and spread of drug (5–7) and diagnostic resistance(8, 9), and to identify patterns of connectivity between parasite populations impacting control efforts (10–13). Aside from genomic applications associated with phenotypic signatures in the parasite populations, such as drug or diagnostic resistance, many applications of malaria genomics rely on identifying levels of parasite relatedness (14, 15). More closely related parasites are suggestive of infections that are closer together in a transmission chain (16, 17). This information is particularly relevant for investigating spatial and temporal patterns of transmission or possible introduction events of parasites into populations.

Genetic relatedness is a measure of shared ancestry, and is often estimated using the concepts of identity by state (IBS) or identity by descent (IBD). Two genomes are identical by state at a particular locus if they have identical genetic sequences. Two genomes are identical by descent (IBD) at this locus if they are IBS and the segment of DNA was inherited from a common ancestor without recombination (16, 18). The overall relatedness between two genomes can be defined as the proportion of loci that are identical by state or by descent, with closely related genomes generally having a higher proportion of identical loci both by state and by descent (19, 20). While IBS metrics are straightforward to calculate using observed sequences, estimating IBD is complicated by the need to account for the possibility that these observed sequences are identical by chance rather than inheritance (16–19).

Estimating the relatedness of *P. falciparum* parasites in two infections using IBD metrics requires minimising the potential role of chance in observing sequences with identical genotypes. To minimise this possibility with a level of precision that can be used to understand transmission dynamics and patterns, multiple long segments of the *P. falciparum* genome are desirable for analysis (21–24). Currently, however, the most widely used sequencing platforms limit the size of DNA fragments that can be genotyped (25) and are further limited by the quality of DNA to be extracted, with longer reads requiring higher quality samples. Estimating IBD with shorter reads requires phasing, i.e., stitching together shorter genotyped segments into contiguous sequences. However, phasing methods for short-read sequences have not been validated for infections consisting of a high number of genetically distinct parasite clones (greater than 3 distinct clones) since it is difficult to identify which segments come from the same clone, and the efficacy of these methods is dependent on the within-host frequency of these clones (26). Therefore, IBD based metrics of relatedness are not well suited to settings of moderate to high malaria transmission where highly complex infections are the norm and longer reads cannot be consistently obtained (27–32).

It is more difficult to determine common ancestry from measures of identity by state, however, statistical methods are frequently used to attempt to indirectly account for the role of randomness in acquiring identical genetic sequences alongside IBS measures (10, 12, 33–35). In the context of using genetic relatedness to determine the level of parasite connectivity between two populations (often across two locations or two timepoints), these statistical methods often aim to test the null hypothesis that there is no clear pattern of connectivity between populations. This is done by repeatedly permuting the labels of the observations (i.e., which population they belonged to) and recomputing the IBS similarity metric, producing a distribution of this statistic under the null hypothesis of these populations being well mixed (12). The determination of whether or not connectivity exists between two populations is usually made on the basis of how extreme the observed relatedness is relative to this null distribution. This method quantifies the strength of evidence for patterns of connectivity. However, when the null hypothesis is not rejected, it is not clear how much this reflects a true lack of effect and how much this is due to limitations of the study design, sample sizes, or relatedness metrics. Less work has been done to better understand the statistical power of these analyses and in particular the possible impact of missing or unsampled infections on estimates of relatedness using IBS-based measures of similarity.

Measures of relatedness, particularly when applied to parasite connectivity, form the core of many applications for integrating genomic data into disease surveillance. Understanding how measures of genetic relatedness behave under different sampling and transmission scenarios is an important component translating genomic data into information about transmission dynamics and patterns that can, in turn, guide optimal malaria control strategies. This work adds to the current understanding by exploring observed patterns of parasite population structure in a more densely sampled cohort than has been previously used and subsequently using a simple set of simulated scenarios to understand how measures of relatedness are impacted under different sampling, and transmission scenarios. We first use data from a longitudinal cohort in a moderate transmission setting in Western Kenya (36) to investigate the spatiotemporal patterns of population structure that can be seen with densely sampled parasite genomic data. We then calibrate an simple agent-based model of malaria transmission with this data and investigate the power using of genomic data to detect spatially-structured patterns of transmission under different sampling schemes, levels of missing data, and transmission intensities in a similar setting. We consider cases where data is missing completely at random, by symptom status to simulate passive case detection, and by complexity infection to simulate other potential biases in sampling. We use two pairwise metrics of genetic similarity, one based on proportion of genetic material shared and one based on the probability of the observed genotype sharing occurring by chance.

## Methods

### Cohort data

A longitudinal cohort of 38 households in Bungoma County, Kenya, a region of high malaria transmission (37, 38) was followed from June 2017 to July 2018. All household members 1 year of age and older were eligible for enrollment with a total of 268 individuals in the cohort. Households were in one of three nearby villages: Kinesamo (*n* = 80), Maruti (*n* = 73), Sitabicha (*n* = 86). On average, households in Kinesamo are 11 km away from those in Sitabicha and 5 km away from those in Maruti, and households in Maruti and Sitabicha are 7 km apart. Dried blood spots (DBS), demographic and behavioural information including questions about recent travel were collected from participants monthly. In addition to regular monthly sampling, symptomatic visits were conducted with participants at the time of reported symptoms consistent with malaria infection where the same information and DBS were collected (see Supplementary Figure S1 for sampling scheme). Treatment was offered for individuals with a positive RDT result. Further details of the sampling and laboratory procedures were previously published (36, 39). Genomic DNA was extracted from the DBS and tested for *P. falciparum* with a duplex TaqMan real-time PCR targeting the *P. falciparum* Pfr364 motif and the human B-tubulin gene.

As previously reported in Sumner et al. (2021), a total of 902 asymptomatic infections were identified over 2,312 monthly visits and a total of 137 symptomatic infections were identified across 501 symptomatic visits. Those positive for *P. falciparum* by quantitative real-time PCR were sequenced at the genes encoding the apical membrane antigen-1 (*Pfama1*) and the circumsporozoite protein (*Pfcsp*) using an Illumina MiSeq platform. Further details on PCR, sequencing, read filtering, and haplotype calling can be found in (39). The study was approved by the ethical review boards of Moi University (2017/36), Duke University (Pro00082000), and the University of North Carolina at Chapel Hill (19–1273). As reported in Sumner et al. (2021), multiplicity of infection (MOI) defined as the number of distinct *Pfcsp* or *Pfama1* haplotypes (each locus is considered separately due to the inability to phase these two segments in polyclonal infections) in each infection was generally high, but similar, across the three villages in this cohort (see Supplementary Figure S2). The overall haplotypic diversity across the infections sampled in the cohort was also high with 209 unique haplotypes identified at the *Pfama1* locus and 155 unique haplotypes identified at the *Pfcsp* locus after filtering (procedure and similar results described in Sumner et al. 2021). The frequency distribution of haplotypes at each of these loci is shown in Supplementary Figure S3.

### Metrics of genetic relatedness

The genome-wide probability of two parasites being identical by descent (IBD) is the gold standard for measuring genetic relatedness of infections since it is the result of inheritance from a common ancestor. However, this metric requires genotyping data from several longer segments of parasite DNA to be accurately calculated. Oftentimes, particularly when smaller segments are sequenced, other measures of IBS-based similarity are used to approximate relatedness, such as the count or proportion of shared alleles between infections. Hypothesis tests for evaluating the strength of evidence for certain hypotheses are particularly useful when using IBS-based metrics, given the possibility of sharing genetic material by chance. When evaluating the level of structure across populations, permutation tests can be used as described below.

First, we propose the following two metrics to estimate the observed genetic similarity and approximate relatedness across pairs of infections. To define the first measure, we first define the asymmetric function of two infections, $\bar{r}$, as the proportion of haplotypes in the first infection that are also found in the second,

(1) $$\bar{r}\left(\mathrm{infection}\;1,\mathrm{infection}\;2\right)\;=\;\frac{\mathrm{number}\;\mathrm{of}\;\mathrm{haplotypes}\;\mathrm{that}\;\mathrm{occur}\;\mathrm{in}\;\mathrm{both}\;\mathrm{infections}}{\mathrm{number}\;\mathrm{of}\;\mathrm{haplotypes}\;\mathrm{in}\;\mathrm{infection}\;1}.$$

We can then define the symmetric measure r as the average in both directions,

(2) $$\begin{array}{rl} r\left(\mathrm{infection}1,\mathrm{infection}2\right)\; & =\;\frac{1}{2}(\bar{r}\left(\mathrm{infection}1,\mathrm{infection}2\right)\;\\ & +\;\bar{r}\left(\mathrm{infection}2,\mathrm{infection}1\right)). \end{array}$$

This metric encodes the expectation that more related infections are more likely to have haplotypes in common while adjusting for the fact that infections with a higher number of clones have more chances to share haplotypes with other infections. The estimate, $\bar{r}$, produces values between 0 and 1 with values closer to 1 indicating higher possible levels of relatedness between infections.

This approximation of relatedness is similar to the Jaccard IBS metric (40), which in this case, would be defined as the number of haplotypes in common divided by the number of distinct haplotypes across both samples. The two metrics generally produce similar results but the proposed metric, r, is higher when the set of haplotypes in one sample is entirely or almost entirely contained in the set of haplotypes in the other (see Supplementary Material for more detail).

The second metric, d, is derived by considering the likelihood that different numbers of shared haplotypes would have occurred in a situation where haplotypes in infections were drawn randomly from the general parasite population. In more detail, for infections 1 and 2 with MOI values $m_{1}$ and $m_{2}$ that share k haplotypes, the metric is defined as the probability that sets of haplotypes of size $m_{1}$ and $m_{2}$ drawn randomly from all observed haplotypes would share at least k haplotypes. The probability of sharing *exactly k* haplotypes can be written as

(3) $$P\left(\mathrm{sharing}\;\mathrm{exactly}\;k\;\mathrm{out}\;\mathrm{of}\;m_{1},\;m_{2}\right)\;=\;\left(\frac{m_{1}}{k}\right)\left(\frac{m_{1}+m_{2}}{m_{2}-k}\right)/\;\left(\frac{m_{1}+m_{2}}{m_{2}}\right)$$

and therefore the probability of sharing *at least* k can be written as

(4) $$\begin{array}{rl} & d\left(\mathrm{infection}\;1,\mathrm{infection}\;2\right)\\ & \;=\sum_{i=k}^{min\left(m_{1},m_{2}\right)}P\left(\mathrm{sharing}\;\mathrm{exactly}\;i\;\mathrm{out}\;\mathrm{of}\;m_{1},m_{2}\right) \end{array}$$

A low probability of sharing a particular set of haplotypes by chance can be interpreted as some evidence of relatedness. This is a measure of differentiation; low values are more likely to be associated with highly related pairs. This measure also gives different values for the comparison of, for example, a pair of monoclonal infections with the same haplotype and a pair of infections with an MOI of 2 with the same two haplotypes. The latter will have a lower value, suggesting less differentiation and higher relatedness. This represents the fact that even for a fixed proportion of shared haplotypes, the greater the number of shared haplotypes between two infections, the more likely the two infections are closely related by a transmission event. This is unlike the first metric, r, or the Jaccard IBS metric, which, in the two cases described above would estimate the same level of relatedness regardless of the absolute number of shared haplotypes.

To obtain expected distributions of, *r,* and *d*, under a null hypothesis of no clear pattern of connectivity between two parasite populations, we randomly permute the population labels (labels can be based on location of origin for the sample, or based on time of sampling) for each infection, and use these permuted labels to estimate the level of structure across these populations. The method for measuring structure across populations depends on the particular question of interest and is detailed below for spatial structure (based on locations of sampling) and temporal structure (based on timing of sampling).

### Population structure by village

If transmission is more likely between individuals in the same village, we would expect to see pairs of infections sampled in the same village to be more related on average than pairs of infections from different villages. We compute the difference between average within and average between village pairwise relatedness and call this the *spatial structure* metric. For village *j* we define the spatial structure as:

(5) $$\begin{array}{rl} \mathrm{spatial}\;\mathrm{structure}\left(j\right)= & \;\frac{2}{N_{j}\left(N_{j}-1\right)}\sum_{k,l\;\in \mathrm{village}\;j\;k<l\;}^{\;}r\left(in\;f_{k},\;in\;f_{l}\right)\;\\ & -\;\frac{1}{N_{j}N_{-j}}\sum_{k\in \mathrm{village}\;j,\;\;l\notin \mathrm{village}\;j\;}^{\;}r\left(in\;f_{k},in\;f_{l}\right) \end{array}$$

where $N_{j},N_{-j}$ are the number of observations within and outside village j respectively. A spatial structure value of 0 would suggest that on average an infection in village j is as related to other infections within village *j* as it is to infections outside village *j*, in other words that there is a high level of parasite relatedness and therefore connectivity between village *j* and other villages. On the other hand, if there is lower connectivity across villages we would expect the spatial structure value to be positive, with a larger value suggesting lower relatedness and therefore lower connectivity. The spatial structure metric was calculated for each of the three villages in the cohort study: Kinesamo, Maruti, and Sitabicha. Similarly, we defined the *spatial differentiation metric* with the same formula as above, replacing the metric *r* with the differentiation metric d. We would expect spatial differentiation to be negative where there is high relatedness between parasites across villages and a high level of connectivity across villages.

A permutation test was used to evaluate the strength of this evidence for village-level structure. This was done by randomly permuting the village labels for each individual and recalculating the spatial structure metric for each village. This generates an approximate distribution of this test statistic (spatial structure) for each village under the null hypothesis that the village an individual lives in has no effect on how related their infection is to any other infection, which would suggest a high level of connectivity between villages. This hypothesis can then be tested by comparing the observed value to this distribution, with the null hypothesis being rejected if it is above a certain percentile in the distribution (such as the 95th percentile). This was repeated for the spatial differentiation metric.

### Structure over time

To investigate whether infections that occurred closer in time were more genetically similar, a linear regression was run on the similarity of each pair of infections r(infection1,infection2) against the absolute number of days between samples being taken. Again, a permutation test was used to test whether the resulting relationship was sufficient evidence to reject a null hypothesis that similarity between infections was unrelated to when they were sampled. In this instance, the dates that the samples were taken were permuted and the linear regression was refit. This produced a null distribution of associations between genetic similarity and time of sampling against which the observed association could be compared. This procedure was then repeated for the metric of differentiation, d.

### Structure between the start and end of the dry season

As an extension to measuring structure over time, we explicitly considered differences by season to identify if there was higher similarity among infections that occurred near the end of the first rainy season (at the start of high transmission) of the study period and beginning of the dry season (from August 1–September 30, 2017) and among infections that occurred at the end of the dry season and beginning of the second rainy season (from March 1–April 30, 2018). If temporal structure exists in general, then we would expect this to be the case in any two time periods separated by many months, however this effect may be larger for these specific time periods as many clones may not persist through the reduction in transmission observed over the dry season in this cohort (36). Here we computed *temporal similarity* and *temporal differentiation* metrics in an analogous way to the spatial metrics defined above, calculating the difference in average pairwise similarity in infections in the same time period and in different time periods. Distributions under the null hypothesis of no temporal structure over these time periods were then obtained by permuting which time period each genotyped infection was in and then recalculating the test statistics.

### Agent based simulation

A simulation model was developed to investigate the impact of study design, sampling schemes, sample sizes, and missing data on the ability to detect population structure under some simplified scenarios. Full details of this model, and the calibration to the cohort based data can be found in (41) and the Supplementary Materials. Briefly, individual-based model of humans (*n* = 200) and female *Anopheles* mosquitoes (stable population of 30,000) was constructed to simulate malaria transmission within our study population. To recapitulate the genetic diversity within and between human infections, as well as some of the transmission dynamics observed in the cohort data, we explicitly simulated infections at the individual haplotype level for a year (365 days) which is roughly the length of the cohort follow up period. We calibrated the model to the cohort data using mosquito and human multiplicity of infection (MOI), and estimates of annual entomological inoculation rate (EIR) (number of infectious bites per person, per year) in nearby areas of western Kenya (42, 43). All genetic data, including estimates of MOI, was calibrated using data from the *Pfcsp* locus.

In order to explore spatial dynamics and clustering under simplified scenarios, the human and mosquito population were split equally into two locations. A proportion (between 0.1 and 0.5) of the population selected at random in each location was eligible to travel for the duration of the simulation, the remainder of the population remained stationary. Travel was only modelled between the two locations in the simulation in order to avoid measuring potential effects of importation on population structure. Mosquitoes did not move between locations, and mosquitoes could only bite people within their designated location. Initially, the human and mosquito populations in each location did not share any haplotypes, however, a burn-in period of 357 days in the simulation allowed some haplotypes to migrate between locations before sampling began. A total of 25 replicate simulations were run for each human travel scenario.

In order to account for the impact of transmission intensity, and haplotypic diversity within infections on various measures of relatedness we also altered some parameters to produce simulation scenarios with lower transmission than the cohort. The distributions of EIR, within-host haplotypic diversity, i.e., mosquito, and human MOI for the low transmission simulations are lower than for the high transmission scenarios (see Supplementary Figure S6).

### Effect of structured missing data

We investigated the effect that structured and unstructured missingness had on the evidence for spatial structure in the relatedness or differentiation of pairs of infections in the simulated data. Given that the majority of studies are likely to sample fewer infections than the cohort, we considered a range of ways that individuals would be subsampled (relative to our sampling). In particular, we considered infections being missed at random in four ways: (1) completely at random (MCAR), (2) missing all asymptomatic infections and subsampling individuals who were symptomatic (to simulate passive surveillance), and differentially by the MOI value with either (3) only if the individual had above median MOI at the time, or (4) only if the individual had median or below median MOI at the time. While sampling by an individual's MOI is not possible *a priori*, missing samples from certain demographics more often (i.e., not MCAR) could have predictable effects on whether high or low MOI infections are more likely to be missed.

The baseline sampling scheme was the same as used in the cohort (see Supplementary Figure S1), monthly sampling of all participants and sampling of symptomatic episodes at any time during the study period. This was modified by missing each of these observations with probabilities 12.5%, 25% or 50% in the missing completely at random scenario and 25%, 50% or 75% in the missing high or low MOI scenarios. In each of the high and low MOI scenarios, these probabilities only applied to around half of the total observations, so the absolute number of observations missing in these scenarios was similar to the missing completely at random scenarios.

We also evaluated the impact of missingness in a passive surveillance scheme (only among individuals who were symptomatic), considering 25%, 50%, and 75% missingness among symptomatic infections, assuming asymptomatic infections remained entirely unobserved.

Each random subsample was repeated five times for each of the 25 simulations used to carry out the permutation test for a total of 125 different datasets to calculate genetic similarity or differentiation under each sampling scenario.

## Results

### The parasite population from a longitudinal cohort in Western Kenya reveals temporal structure, but no spatial structure

**Figure 1:**
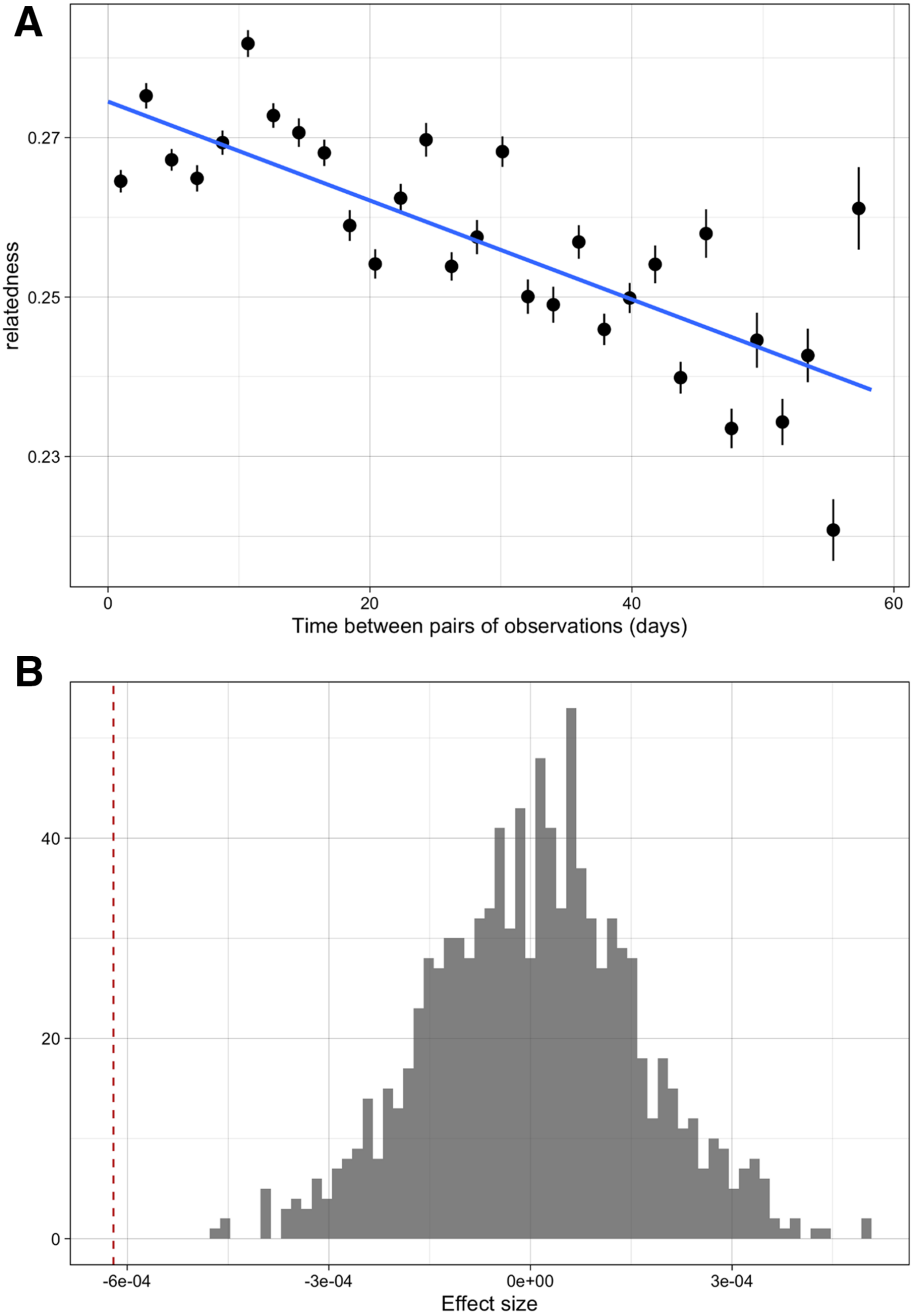
The overall patterns of relatedness across time amongst samples from the cohort data. (**A**) Relationship between pairwise relatedness and time between samples. Points represent averages of pairs of observations similar numbers of days apart. (**B**) The effect of time between infections in weeks on relatedness (red dashed line) compared to a null distribution where there is no temporal structure to relatedness (grey histogram).

**Figure 2:**
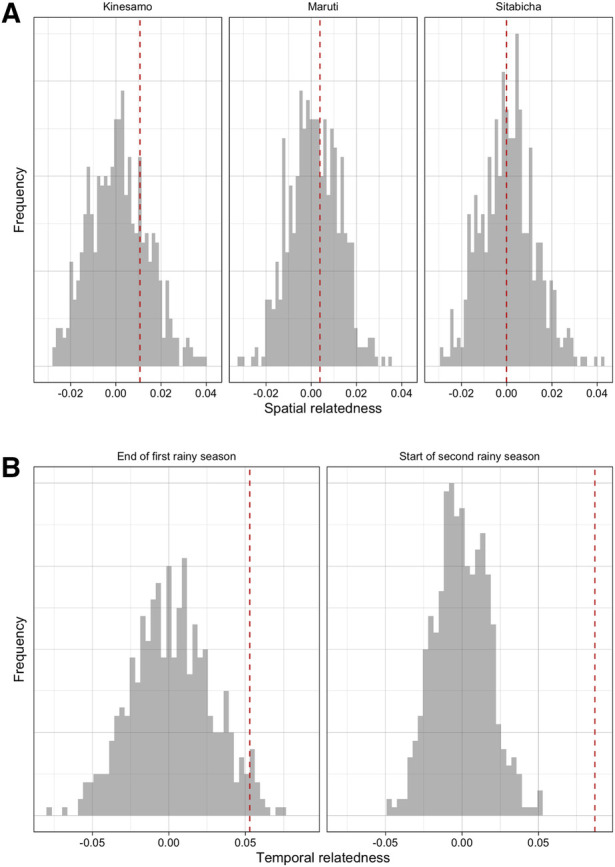
The overall patterns of differentiation across space and time amongst samples from the cohort data. (**A**) Spatial relatedness in cohort data in each village (difference in mean relatedness in pairs of infections where both are from the specified village and pairs where only one is from that village). (**B**) Temporal relatedness (difference in mean relatedness in pairs of infections where both are from the same time period and pairs from different time periods) when considering the end of the first rainy season and start of the second rainy season. In both panels, the value observed in the data (red dashed line) is compared to a null distribution where there is no village or temporal structure, respectively (grey histogram).

Over the course of the study period, infections that were sampled closer in time were more likely to be genetically similar than those sampled further apart. Time between samples and genetic similarity (quantified by the metric r) were negatively associated (Figure 1A); when a linear model of relatedness by time apart was fit, a one day difference in infection observation dates corresponded to an average difference in genetic similarity of of 8.86 × 10^−5^. While this effect was small it was statistically significant, as this value was lower than all values generated under the null hypothesis of no temporal structure (Figure 1B). Similarly, infections sampled closer in time were less differentiated (quantified by the differentiation metric d). Again this effect was small (on average a 1.05 × 10^−4^ increase in differentiation per day apart) but statistically significant (Supplementary Figure S7). If we discretize the data and only consider seasons (first and second rainy seasons), we do see some additional evidence of temporal relatedness, with pairs of infections from the same time period having higher similarity and lower differentiation on average than pairs that spanned both time periods. This temporal similarity was 0.052 and 0.087 for the two time periods, respectively, and the temporal differentiation was −0.017 and −0.041. These values for genetic similarity (metric, r) were around the 97 and 100th percentiles of the null distribution but for differentiation (metric, d) they were only around the 19th and 6th percentile (Figure 2B, Supplementary Figure S8). Despite having a period of low transmission, which could theoretically result in a genetic bottleneck of the parasite population, we did not see strong statistical evidence for these comparisons possibly due to the smaller number of comparisons or the overall sustained moderate levels of transmission (see Supplementary Figure S9).

In comparison, there was little evidence of village-level structure in genetic similarity among infections sampled in the longitudinal cohort. In Kinesamo and Maruti, infections within the village were on average slightly more related to others within the same village compared to elsewhere, with spatial similarity values of 0.01 and 0.0038, while in Sitabicha there was little difference (spatial relatedness of −7.46 × 10^−5^). These values were around the 75th, 63rd and 45th percentiles of their respective distributions under the null hypothesis of no village-level structure or high connectivity between villages (Figure 2A) and therefore there was little evidence to reject this null hypothesis. The spatial differentiation metric also showed little evidence of village-level structure (Supplementary Figure S8). Restricting the pairs of infections used to those observed less than 21 days apart, infections that should be more likely to be related by transmission, produced similar results (Supplementary Figure S10). Additionally, extremely low Fst and Jost's D statistics computed based on observed *Pfcsp* and *Pfama1* haplotype frequencies across the three villages further corroborate the lack of evidence in these data for population structure at the village level (See Supplementary Figure S11).

### Investigating the lack of spatial structure with a simplified agent-base model of transmission

To further explore the lack of village-level structure in relatedness or differentiation of infections in cohort data, we constructed a simplified agent-base model of malaria transmission amongst a population similar to our cohort (see Supplementary Materials). There are multiple factors that could result in a lack of structure, some of these mechanistic in nature and others *via* our observation process. For example, particularly in higher transmission settings with high parasite diversity, the metrics used may not be sensitive enough to observe differences in such complex infections. Moreover, movement between two populations can result in less structure between locations with infections being both imported and acquired *via* infected travellers. Finally, the observation process including who is sampled, at what frequency, and what types of infections could result in an underpowered analysis. While it is not feasible to investigate all of these possible factors in the data directly, with the simulation model we generated individual human infection histories in two locations to evaluate how our metrics of similarity are impacted by various scenarios comparing these possible causes. We varied levels of mixing, transmission intensity, and sampling to explore how differentiation and genetic similarity metrics performed in these simulated populations with a focus on rejecting the null hypothesis of no structure or high connectivity with high enough probability.

**Figure 3:**
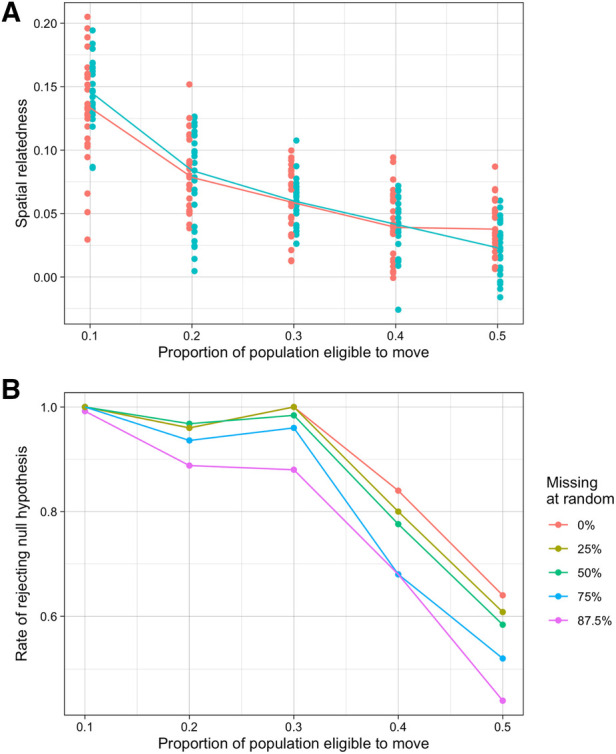
Simulated results of spatial relatedness under various mobility and missingness conditions. (**A**) Spatial relatedness across multiple simulations and different proportions of the population moving between locations. Values for each location are shown in different colours and the median is shown by the line. (**B**) Rate that the null hypothesis of no location relatedness structure was rejected at different proportions of random missingness.

Here, we present results of between-infection relatedness across the two locations in our simulation model under different levels of mixing between the populations. We allowed between 10% and 50% of the population in each location to be mobile (i.e., could ever take a trip in the other location) and set the probability of taking a trip to the other location for these individuals in the model at any given day to be 0.01. While we considered lower values for this probability, a probability of 0.01 best matched the distribution of trips recorded in the study (Supplementary Figure S5). This is equivalent to around 3 trips a year by each individual who was mobile. The first sampling scheme we considered approximates the sampling carried out in the real study (see Supplementary Figure S1). Under this sampling scheme, as the proportion of the population able to move increased from 10% to 50% genetic similarity between locations decreased from around 0.1 to less than 0.025 on average (Figure 3A). This statistic was also highly variable between different simulation iterations (25 in total for each scenario) relative to the values of genetic similarity between locations. When smaller proportions of the population were eligible to move, the null hypothesis of no spatial structure was almost always rejected suggesting lower connectivity, however when 40% and 50% of the population were able to move the rate of rejecting the null hypothesis (essentially the power of the hypothesis test) fell to 0.84 and 0.64 respectively (Figure 3B). These results were similar for the differentiation metric, though power was slightly lower when mixing was high (Supplementary Figure S12).

### The impact of sampling and transmission intensity on inferring relatedness

We also assessed how different kinds of missing data affected the evidence for spatial structure or connectivity. Missing symptomatic and asymptomatic infections completely at random did not change average estimates of spatial relatedness (i.e., did not introduce bias). As expected, as missingness increased, we saw greater variation in these estimates in each simulation across the different sampling repeats (125 total datasets), resulting in lower rates of rejecting the null hypothesis. When up to 50% of observations were missing, this reduction in power was relatively small, at most reducing from 0.64 with no missing data to 0.58 with 50% missingness when half of the population were mobile. At higher rates of missingness, however, the power decreased substantially. For example, when 87.5% of observations were missing the power when half of the population were mobile was around 44% (Figure 3B, Supplementary Figure S13).

We further explored additional biases such as only sampling symptomatic infections (passive sampling) which might be a more feasible way to sample a large number of infections than capturing both asymptomatic and symptomatic infections as we have in the cohort. Only sampling symptomatic infections (passive sampling) did not introduce any bias and generally had a similar effect on the power as missing the same proportion of infections under the cohort's active sampling scheme (Supplementary Figure S15). We further explored other types of infections that may be differentially sampled such as by MOI and found that including more complex infections in the analysis did result in higher estimates of spatial relatedness (see Supplementary Figure S14).

**Figure 4:**
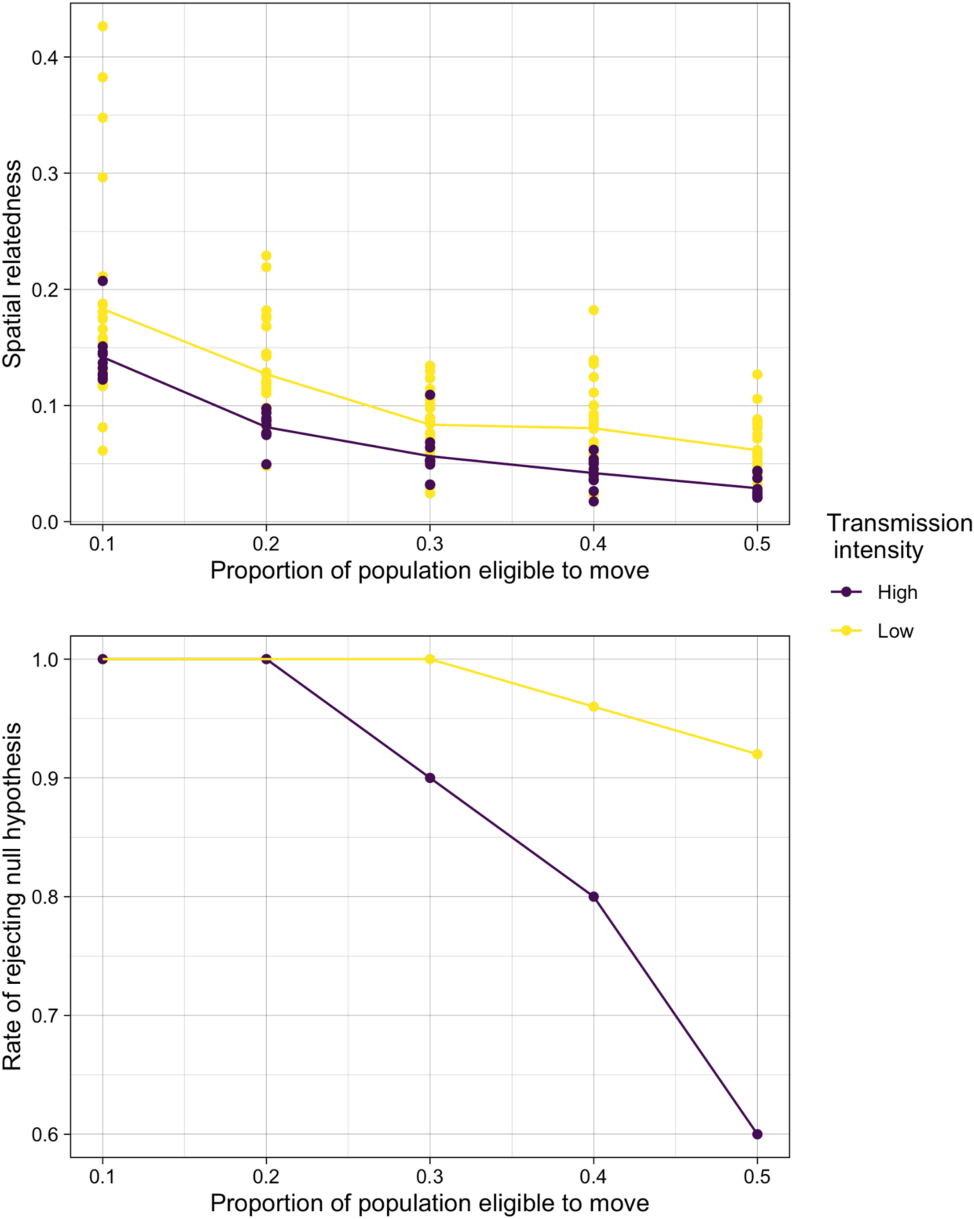
The comparison of relatedness results in simulated high and low transmission settings. (**A**) Spatial relatedness (averaged across both locations) and (**B**) rate of rejecting the null hypothesis under normal sampling in the high and low transmission intensity settings.

Finally, we explored the impact of high transmission (and corresponding high parasite diversity) could also impact our ability to detect spatial structure in the population. We explored lower transmission (and corresponding lower parasite diversity) in the simplified simulation model and compared these results to our high transmission scenarios. As expected, in a lower transmission setting, we found that estimates genetic similarity were consistently higher than in the original simulations reflecting the higher transmission setting of the cohort (Figure 4). Furthermore, this increased evidence of spatial structure resulted in much higher rates of rejecting the null hypothesis for high levels of mixing. When 50% of the population were able to move, for example, the null hypothesis was rejected more than 90% of the time in the low transmission scenario compared to 64% in the higher transmission scenario.

## Discussion

In this study, we evaluated *Plasmodium falciparum* genetic similarity and differentiation across geographic location and time in a longitudinal cohort from a region of high malaria transmission region of Western Kenya. Using IBS-based metrics, we observed statistically significant temporal structuring. Since we considered changes in relatedness over a continuous timescale, the effect size was predictably small, but the level of statistical significance suggests that there are temporal changes in the genetic composition of the parasite population which merit further investigation to evaluate the degree to which these genetic changes could be evidence of population bottlenecks across transmission seasons. Conversely, we found no evidence of spatial structure in the data. To explore factors that may have contributed to the lack of spatial structure, we constructed a simplified simulation study of small villages to evaluate the impacts of connectivity, transmission, and sampling on measures of genetic similarity and differentiation. We found that a study design similar to that of the cohort was able to detect spatial structure in the simulated data at low levels of connectivity across two locations with high power. However, as the connectivity increased and higher rates of missingness were introduced in these data, the power to detect spatial structure decreased sharply. This was despite only moderate decreases in the average value of the test statistic – the difference between within and between location pairwise relatedness, as measured by IBS metrics of genetic similarity and differentiation. Then, using the simulation model, we investigated how these results translated to a hypothetical setting with lower malaria transmission. In general, the decrease in power under higher rates of missingness and connectivity was far less pronounced in the low transmission scenarios.

Together, these results suggest that the active sampling scheme employed in the cohort can detect spatial structure under many scenarios and is quite robust to different patterns of missing data. However, in high transmission settings in particular, the sample sizes required to reject a null hypothesis of no spatial structure across areas with similar observed levels of genetic similarity or differentiation may be vastly different, since the drop off in power was quite steep for moderate to high levels of connectivity. Furthermore, in these high transmission settings, even intensive, active sampling, may not be sufficient to detect spatial structure under moderate to high amounts of connectivity. More sophisticated IBD-based metrics that make efficient use of genetic data may be ideal in these settings, however, these methods remain difficult to employ among infections with high within-host genetic diversity.

Our model makes several simplifying assumptions about malaria transmission and is heavily informed by the observations from the cohort study in western Kenya. However, by exploring some extreme models of parasite connectivity and sampling schemes, we were able to infer certain general trends about the likelihood of detecting spatial structure under different scenarios. Furthermore, if no spatial structure was detectable under these more extreme scenarios, it is unlikely that this structure would be detectable in other real-world scenarios. Interestingly, under scenarios where the probability of sampling an individual was conditioned on the MOI of their infection, we found that sampling more complex infections with a higher probability resulted in higher estimates of spatial relatedness. While it is not possible to design sampling schemes based on MOI *a priori*, there are several factors known to be associated with differences in MOI, such as age and transmission intensity (44, 45). Therefore, sampling schemes that are more likely to miss certain age groups, such as household sampling that takes place while children are at school, or sampling strategies that tend to miss adults who routinely travel to regions of differing transmission intensities, may systematically miss high or low MOI infections. Furthermore, independent of possible sampling schemes, differences in MOI have been shown to be associated with differences in transmission intensity (44, 45). Therefore, understanding how measures of genetic similarity or differentiation are impacted by within-host diversity is an important component of understanding how parasite connectivity can be measured across a wide range of malaria endemic areas. Many studies have revealed that asymptomatic infections contribute disproportionately to the infectious reservoir (39, 46–49), therefore, we investigated whether missingness based on symptom status impacted our inference of parasite population structure and connectivity. We found that passive sampling schemes where only symptomatic infections were captured did not bias estimates of genetic similarity or differentiation.

There are several limitations to this study, most importantly, while every effort was made to calibrate the model to data from a natural setting, it falls short of replicating reality in several ways. First, we only allow travel between two locations where genetic data on parasite populations was available from both locations. In reality, human movement is far more complex, and in most circumstances, it is not possible to obtain parasite genetic data from all visited locations where infections may be acquired or lead to secondary transmission. These gaps can obscure patterns of spatial structure in ways that we did not account for in our study. Additionally, we did not allow for mosquito movement between locations which may also be an important driver of connectivity on small spatial scales, and therefore would be an important component of transmission to evaluate. Furthermore, this model simulates two populations of 100 individuals each therefore, we are unable to investigate how our findings about the ability to reject a null hypothesis of no spatial structure may scale to much larger population sizes, such as counties or other administrative units in Kenya, which may be more relevant for control programs. However, the use of malaria genomics to explore connectivity may be most relevant in low transmission settings where transmission can be focal, and therefore interventions are deployed on finer spatial scales. In this same vein, correlation structures that arise at the household level and among individuals with repeated measurements were not accounted for in the model. In previous analyses (41) we did not find strong evidence of household correlation in this cohort study however, other have found evidence of household structure (50). We also did not model individual level correlation structures which could arise from individual immunity to particular parasite clones making reinfection or symptomatic episodes with certain haplotypes more or less likely and impacting relatedness over time and across space. Some more sophisticated models do accomplish this (51), and previous studies of this cohort found differences in the likelihood of symptomatic infection in infection events with only haplotypes that had not previously been detected in that individual. However this same analysis failed to find clear patterns in the probability of symptomatic infection when both previously detected and new haplotypes were found and did not find any clear trends in the probability of reinfection with particular haplotypes making it difficult to use these data to inform a model individual-level immunity (52).

Additionally, we calibrated the model to genetic data from a single locus in the *P. falciparum* genome, adapting our proposed measures of relatedness to incorporate information across more loci and evaluating the effects of added genetic information would be an important expansion since oftentimes more than one locus is genotyped in a study. Moreover, we tested only two extreme levels of transmission in study; future analyses that explore a wider range of transmission scenarios, particularly low transmission settings, and that are calibrated to detailed epidemiological and genomic data from a range of malaria endemic areas would be informative to help control programs determine the appropriate sampling scheme, size, and frequency for malaria genomic studies. Finally, we did not compare our results to those obtained under an IBD metric, this is largely because IBD based metrics have yet to be adapted and validated for the amplicon deep sequencing data available from the cohort study. The majority of these methods have relied on longer reads or whole genome sequencing data that allows for more substantial measures of relatedness to be inferred. However, it would be useful to leverage other datasets that may be better suited to IBD based metrics to directly compare the performance of our IBS based metrics under different sampling schemes to IBD based metrics, since these are a gold standard of measuring genetic relatedness.

Genomic surveillance has the potential to reveal patterns in malaria transmission aiding in effectively targeted control measures. Leveraging *P. falciparum* genetic data for these purposes relies on measuring parasite relatedness. While genetic relatedness measures that are based on IBD remain the gold standard, there are several challenges to adapting these methods for *P. falciparum*. Currently, IBS based measures of relatedness are much more practical to implement, particularly in settings of high transmission where complex infections are common. Therefore, understanding the ability of IBS based measures to detect various levels of genetic relatedness and patterns of connectivity under different sampling schemes, and across different levels of malaria transmission is an important component of designing effective studies and producing data that can be used to inform malaria control strategies.

## Data Availability

The original contributions presented in the study are included in the article/**Supplementary Materials**, further inquiries can be directed to the corresponding author/s.
