# Supplementary material for: Exploring how space, time, and sampling impact our ability to measure genetic structure across Plasmodium falciparum populations
Source: Front Epidemiol. 2023 Feb 17;3:1058871. doi: 10.3389/fepid.2023.1058871 (PMC10956351; doi:10.3389/fepid.2023.1058871)
Supplement: Supplementary file 1 [file Datasheet1.pdf]

Supplementary material

Comparison of relatedness metric to Jaccard IBS metric

When considering samples sequenced at multiple loci or polyclonal infections, a common metric used to quantify IBS is the Jaccard metric. For samples A, B this is defined as

$$|A \cap B| \;/\; |A \;\cup\; B|$$

or in other words, the number of elements in common between samples divided by the number of unique elements across both. In our case, this would be defined as

$$\frac{number\ of\ haploytes\ shared}{number\ of\ unique\ haplotypes\ across\ both\ samples}.$$

For example, the relatedness as measured by this metric between a sample containing haplotypes 1, 2 and a sample containing haplotypes 1, 2, 3, 4, 5 would be 0.4. Our relatedness metric, on the other hand, would quantify the relatedness in this case as the average of 1 (the proportion of haplotypes in sample one also found in sample two) and 0.4 (the proportion in sample two also found in sample one), that is 0.7.

In general, these metrics produce similar results, but in general our metric assigns higher relatedness to cases where the haplotypes in one sample are entirely or almost entirely contained in the other. In some cases this behaviour is desirable - for example it is possible than the second individual in the example above (with a MOI of 5) was directly before the first individual in a chain of transmission, as it is possible [common?] that not all haplotypes were transferred to the mosquito or the second individual. A relatedness metric would ideally produce high values for such closely related infections. However, this also has the potential to produce more “false positives”, i.e. pairs of samples that are assigned relatively high relatedness values despite not being closely connected in transmission chains. Figure S15 shows some more comparisons of relatedness values for the two metrics.

Figure S1: Sampling scheme for cohort study.

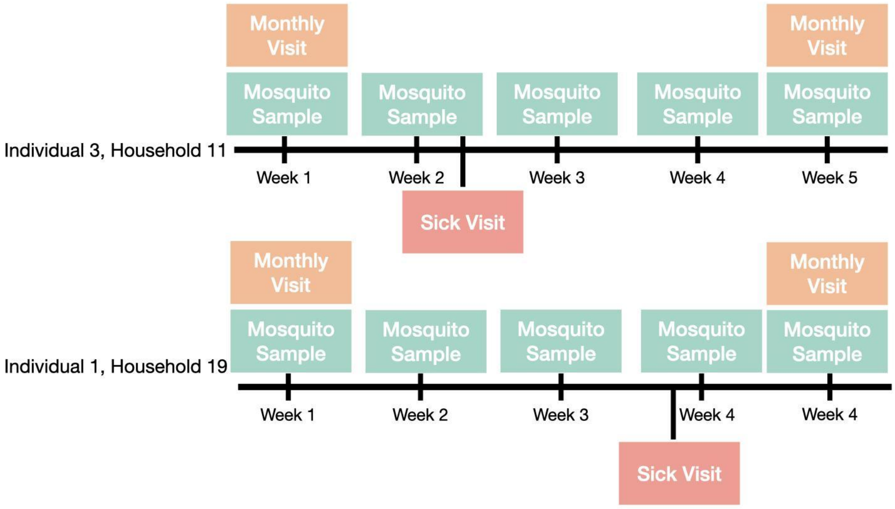

Figure S2: Multiplicity of infection (MOI) at *Pfcsp* and *Pfama1* loci across three study villages.

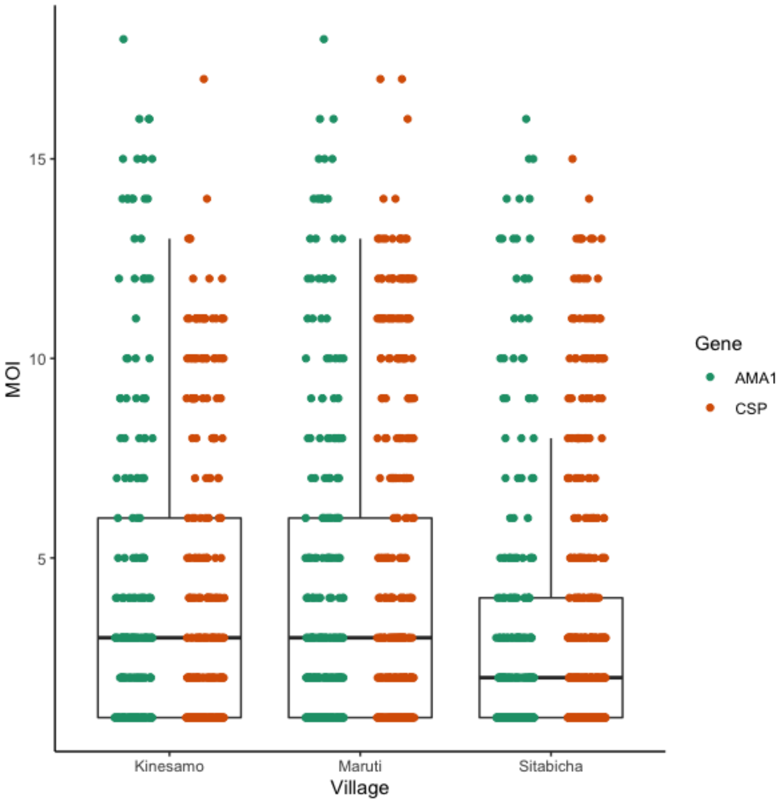

Figure S3: Frequency distribution of *Pfcsp* and *Pfama1* haplotypes in sampled infections.

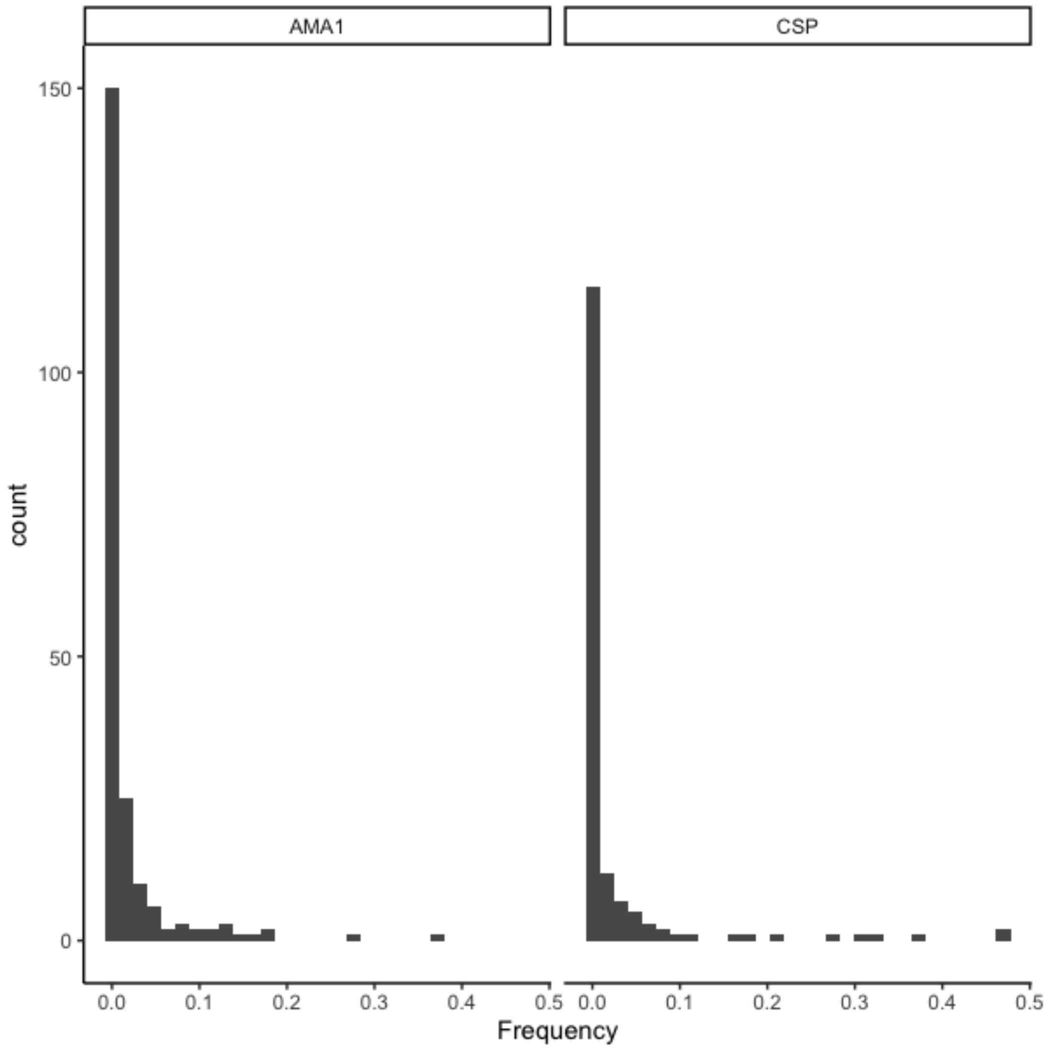

<u>Detailed description of the agent-based model:</u>

Initial conditions: We set initial conditions to broadly reflect those observed in the cohort. Initially, humans are randomly designated as infected with a probability approximately equal to the observed PCR positivity rate at monthly visits excluding sick visits (pr=0.3). If infected, the number of distinct haplotypes in the human infection (multiplicity of infection, MOI) is drawn from a truncated Poisson distribution fit to the data for the *Pfcsp* locus (mean = 2, maximum = 16, see Figure S2). Conditional on the initial MOI for a particular individual's infection, the haplotypes that comprise that infection are drawn from the list of observed *Pfcsp* haplotypes with probability weights equal to the observed population frequency for *Pfcsp* haplotypes (see Figure S3). Finally, the time since a person was initially infected with each haplotype present in their infection is drawn independently from a Poisson distribution with a mean of 20 days. This is to allow a wide range of possible durations of human infections, and independent draws for each haplotype allow for the possibility of superinfection in humans.

The age-distribution of the initial population of 30,000 adult female *Anopheles* mosquitoes are drawn from a truncated Poisson distribution (mean = 4, maximum = 14 days) based on the daily survivorship of female *Anopheles* mosquitoes in the wild (CDC-Centers for Disease Control and Prevention 2009). Conditioned on age, the mosquito is designed as infected or not with older mosquitoes more likely to be infected and have more complex infections. Similar to human infections, the overall MOI distribution matches the distribution found in mosquito abdomens for *Pfcsp* (see Sumner et al. 2021). Like humans, the number of days since the mosquito was initially infected with each haplotype in their infection is drawn independently from a discrete uniform distribution between 1 day and age of the mosquito.

Transmission dynamics: The model is incremented daily for a total of 722 days (357 days of burn-in and 365 days of sampling) where the following transitions can occur (see Figure S4 for graphic representation):

1. Mosquito biting behaviours: Whether or not a mosquito will take a human blood meal is determined probabilistically by the last time they fed. If they fed within the last three days, this is considered an “off day” and their probability of feeding on a human is 0.01, lower than on an “on day”, where the mosquito has not fed within the last three days, then the probability of feeding on a human is 0.1. This setup allows, with relatively low probabilities, for mosquitoes to feed on humans multiple days in a row to complete a single blood meal. This is supported by evidence of female *Anopheles* mosquitoes imbibing multiple blood meals per gonotrophic cycle (Scott and Takken 2012; Thongsripong et al. 2021). This simulation also allows for the possibility that a mosquito may not feed on a human, which can occur if the mosquito feeds on other animals, or does not successfully find a host. Though, in most cases the model assumes that a mosquito will feed on a human at least once in their lifetime, which is supported by the identification of 73% of the collected mosquitoes as *An. gambiae* or *An. funestus*, both highly anthropophilic species (Kabbale et al. 2013; Mbogo et al. 1993). Once a human feeding event has been determined, a discrete probability distribution is used to determine how many individuals the mosquito will feed on during the 24-hour period (1 = 0.6, 2= 0.35, 3=0.04, 4=0.01). All humans have an equal chance of being bitten. The values of mosquito feeding parameters were also determined by calibrating MOI and EIR values to those from the cohort data.
2. Infectious bites: If a mosquito feeds on a human, and the human, mosquito, or both are infectious, a successful transmission event can occur. Haplotypes are eligible to be transferred from a human to a mosquito if they have been in the human for at least 14 days and vice versa if they have been in the mosquito for at least 9 days. Whether each eligible haplotype is transferred during an infectious bite either from human to mosquito or vice versa is determined by independent draws from a Bernoulli distribution (p=0.6 for human to mosquito transfer, p=0.3 for mosquito to human transfer). Within a simulation scenario use the same probability for all haplotypes. These probabilities were determined by calibrating MOI and EIR values to those from the cohort data.
3. Parasite clearance without treatment: In the cohort, we observe multiple instances of participants with asymptomatic infections who are subsequently PCR negative at a following time point without treatment . Additionally, we also observe instances when individual haplotypes were present at one asymptomatic visit, and absent at the next,

while the person remained asymptomatically infected. Based on these observations, we allow for individual haplotypes to be cleared with some probability if the time since infection is 30 days or longer with probability 0.85, which was determined by calibrating MOI and EIR values to those from the cohort data.

4. Human travel: during each timestep the subset of the population eligible for travel is chosen for a trip (if they are not already on one) with probability 0.005, 0.01 or 0.03 depending on the scenario. If the person is selected to travel the duration of their trip is selected from an exponential distribution with a mean of 8 days (taken from the average trip duration in the cohort data which is 8 days), the person remains in the opposite location from their starting location for the duration of the trip. People are not eligible for sampling while they are on a trip. The middle scenario (probability 0.01 of movement) is the scenario that most closely approximates the distribution of the number of trips per mobile person observed in the cohort data (see Figure S5).
5. Symptomatic infections and treatment: Any infection containing at least one haplotype that was introduced in a human 7 or more days prior, can become symptomatic with probability 0.05 which is the observed proportion of successfully genotyped infections that were symptomatic. We assume that symptomatic infections are sampled and treated immediately with all haplotypes in this infection cleared at the time of treatment, and that treatment offers protection from reinfection for 14 days (Bretscher et al. 2020).
6. Mosquito demography: for each mosquito, the probability of mosquito survival on any given day is negatively correlated with age. This relationship was chosen to produce a stable age-structured mosquito population with the maximum survival length to be 2 weeks. As mosquitoes die, they are replaced with newly emerged adult females to maintain a stable population size.

Figure S4: A graphic representation of the steps that take place during a single increment (one day) of the simulation. Complete descriptions of each stage (1-6) are detailed above. Adapted from (Bérubé et al. 2022).

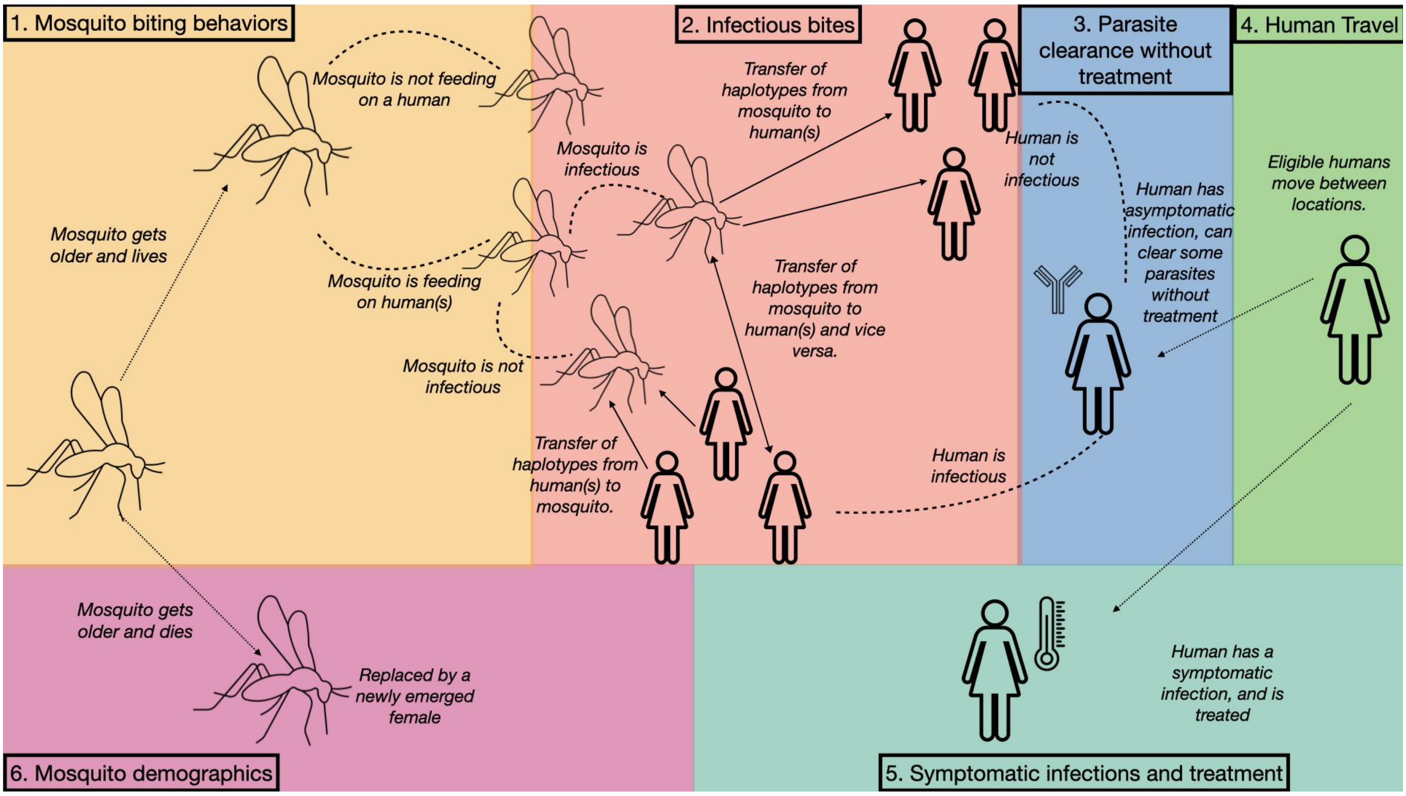

Figure S5: distribution of the number of trips taken by each mobile person annually under different movement scenarios.

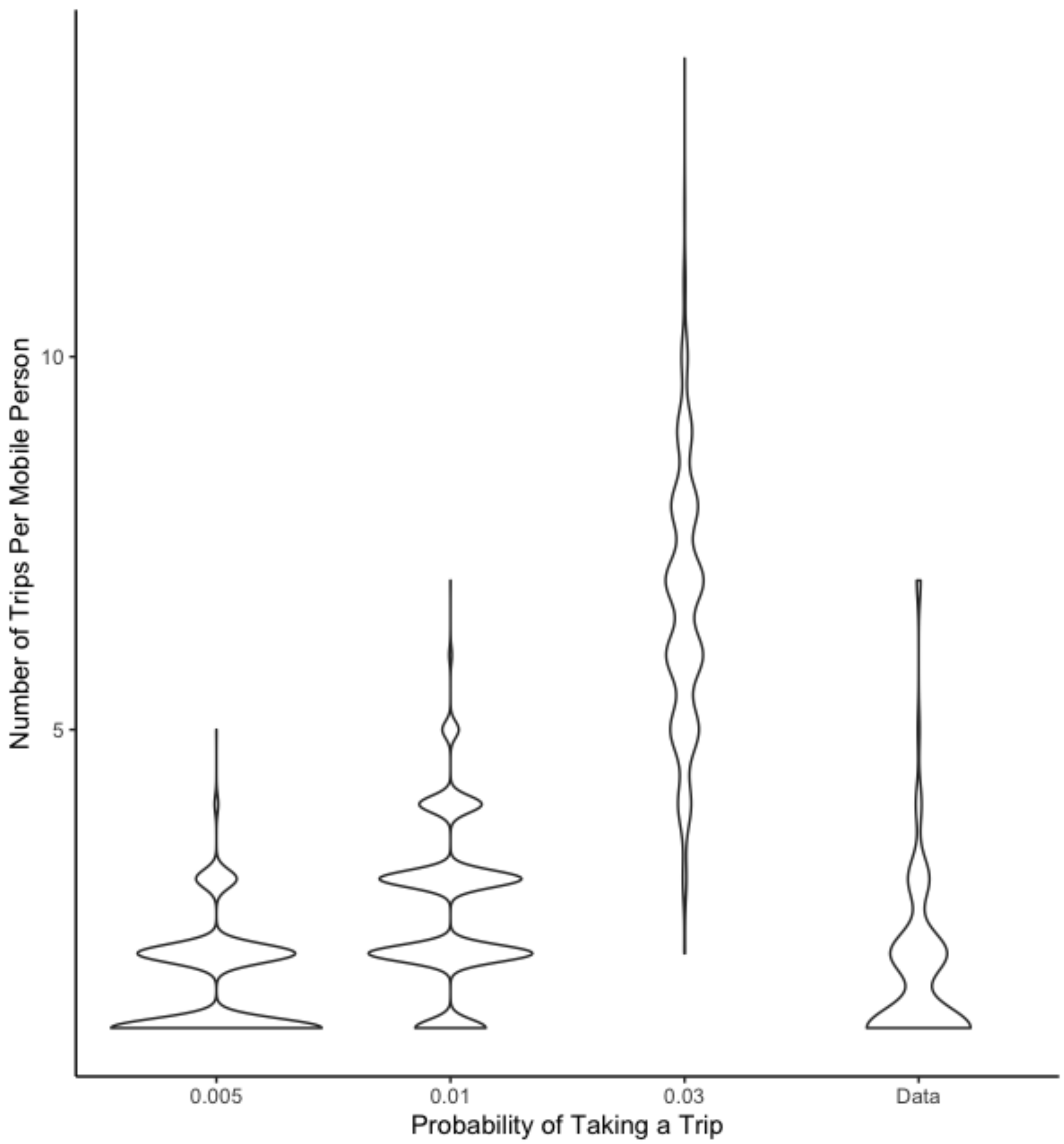

Figure S6: EIR, and MOI distributions for simulations with high and low transmission. Dots show a random subset of 1000 human and mosquito MOI values and EIR estimates across all simulation scenarios, including different levels of movement.

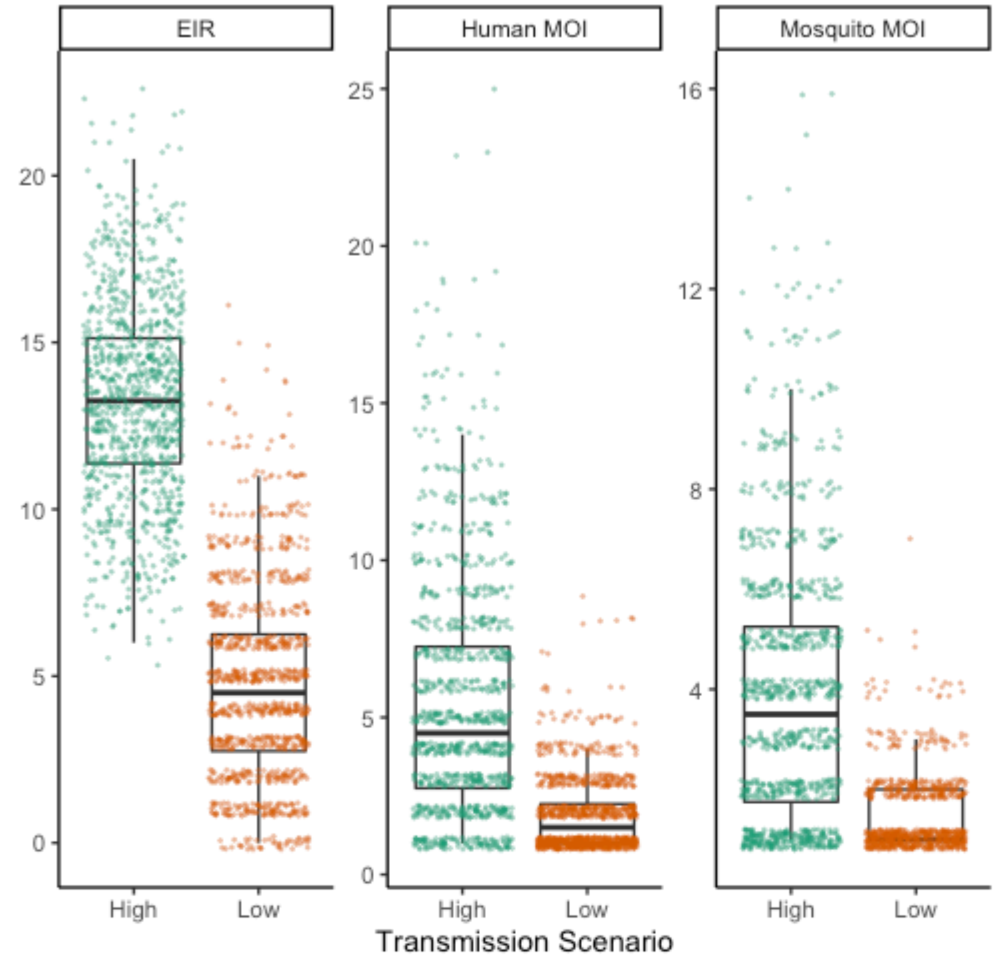

Figure S7: Pairwise differentiation over time. (a) Relationship between pairwise differentiation and time between samples. Points represent averages of pairs of observations similar numbers of days apart. (b) The effect of time between infections in weeks on differentiation (red dashed line) compared to a null distribution where there is no temporal structure to relatedness (grey histogram).

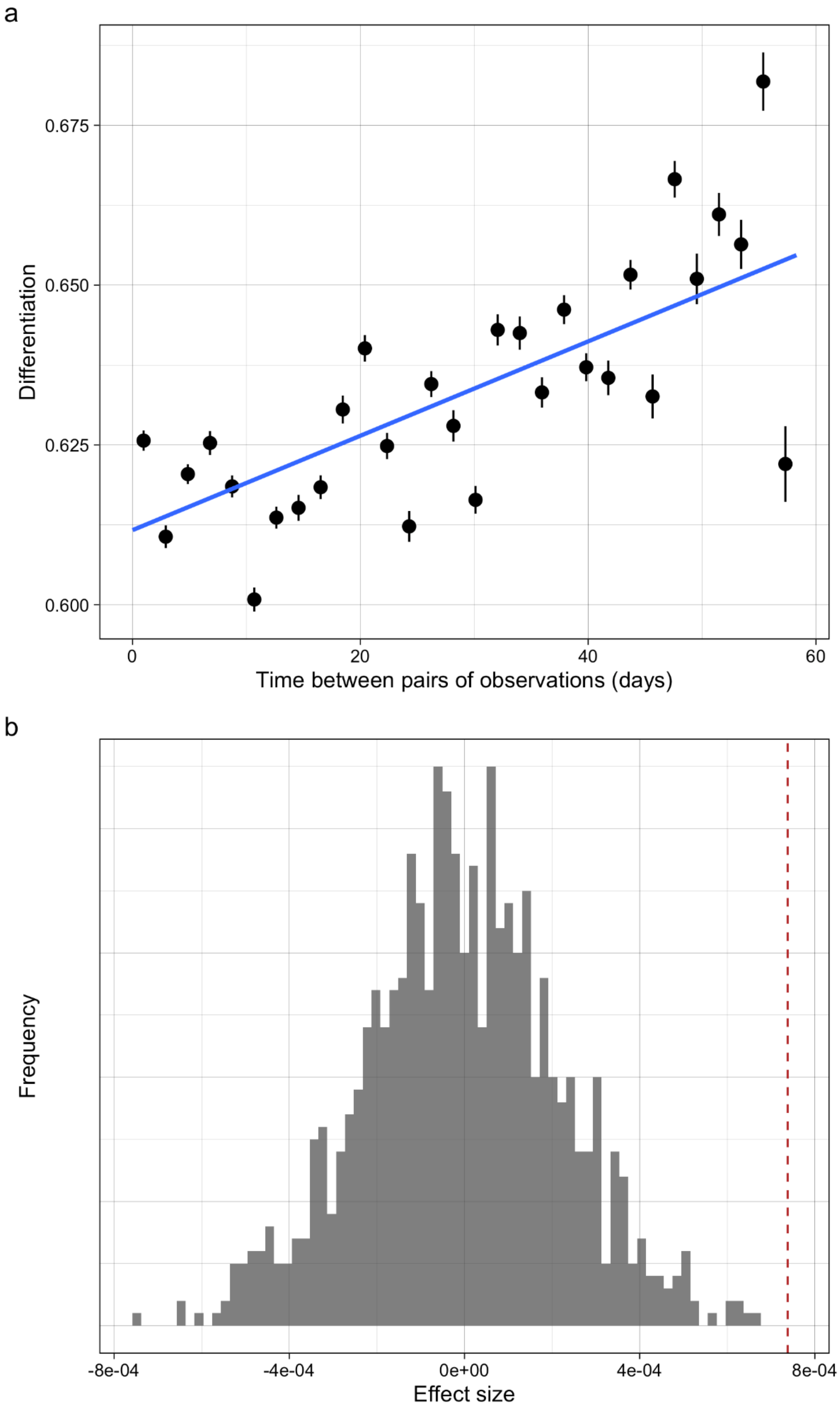

Figure S8: Spatial and temporal differentiation in cohort data. The value observed in the data (red dashed line) is compared to a null distribution where there is no village or temporal structure, respectively (grey histogram). (a) Spatial differentiation in each village. (b) Temporal differentiation in the end of the first rainy season and beginning of the second rainy season.

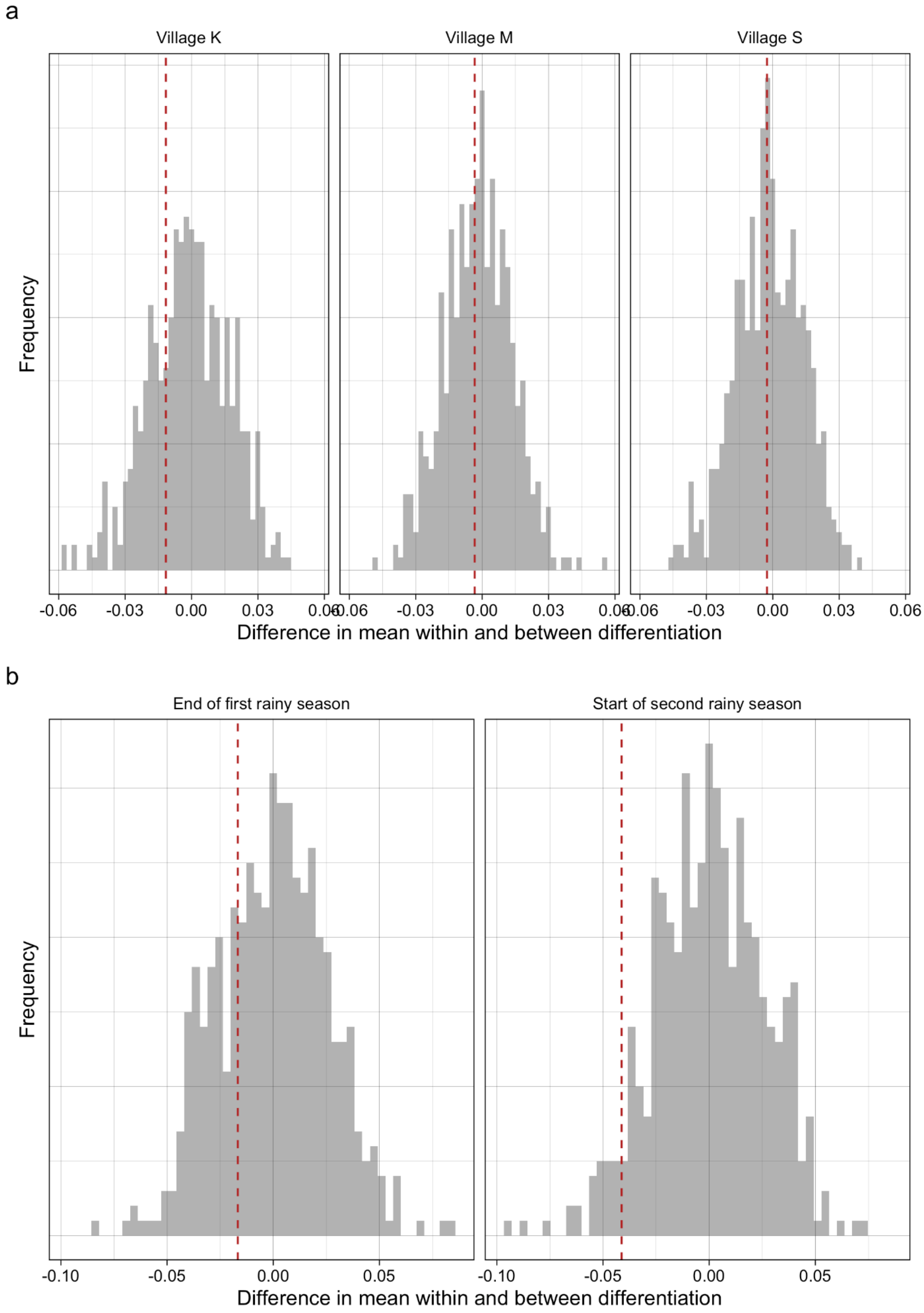

a
Village K
Village M
Village S
Frequency
-0.06
-0.03
0.00
0.03
0.06
Difference in mean within and between differentiation
b
End of first rainy season
Start of second rainy season
Frequency
-0.10
-0.05
0.00
0.05
Difference in mean within and between differentiation

Seasonal differences in the parasite population:
The general haplotypic diversity did not change substantially from the end of the first rainy season and the start of the second rainy season. From the 155 unique *Pfcsp* haplotypes in the human population, 39 were detected at both time periods, 19 were detected at the end of the first rainy season, but not at the start of the second rainy season, and 32 were detected at the start of the second rainy season that were not present at the end of the first rainy season. Overall, the population frequency of each haplotype, defined as the number of infections from each time period with a haplotype divided by the total number of sampled infections in that time period, did not change substantially with the median absolute difference in frequency between the two periods of 0.005 (see figure S9).

Figure S9: Distribution of absolute difference in haplotype frequencies from the end of the first rainy season (August 1, 2017- September 30, 2017) to the beginning of the second rainy season (March 1, 2018,- April 30, 2018).

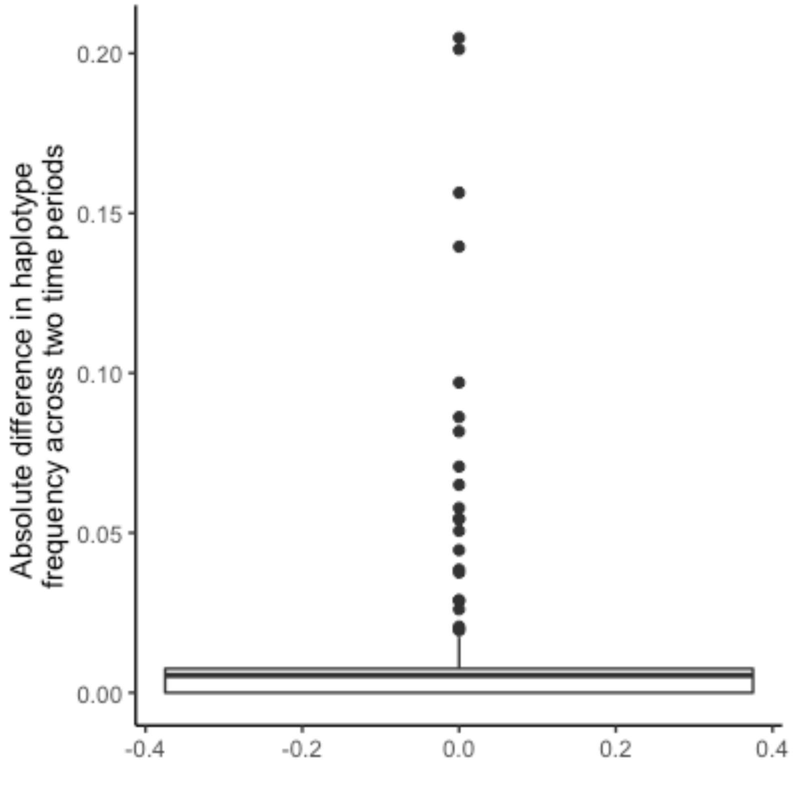

Figure S10: Spatial relatedness (top) and differentiation (bottom) in cohort data in paris of infections observed within 21 days of each other. Observed data (red dashed line) and null distribution with no spatial structure (grey histogram).

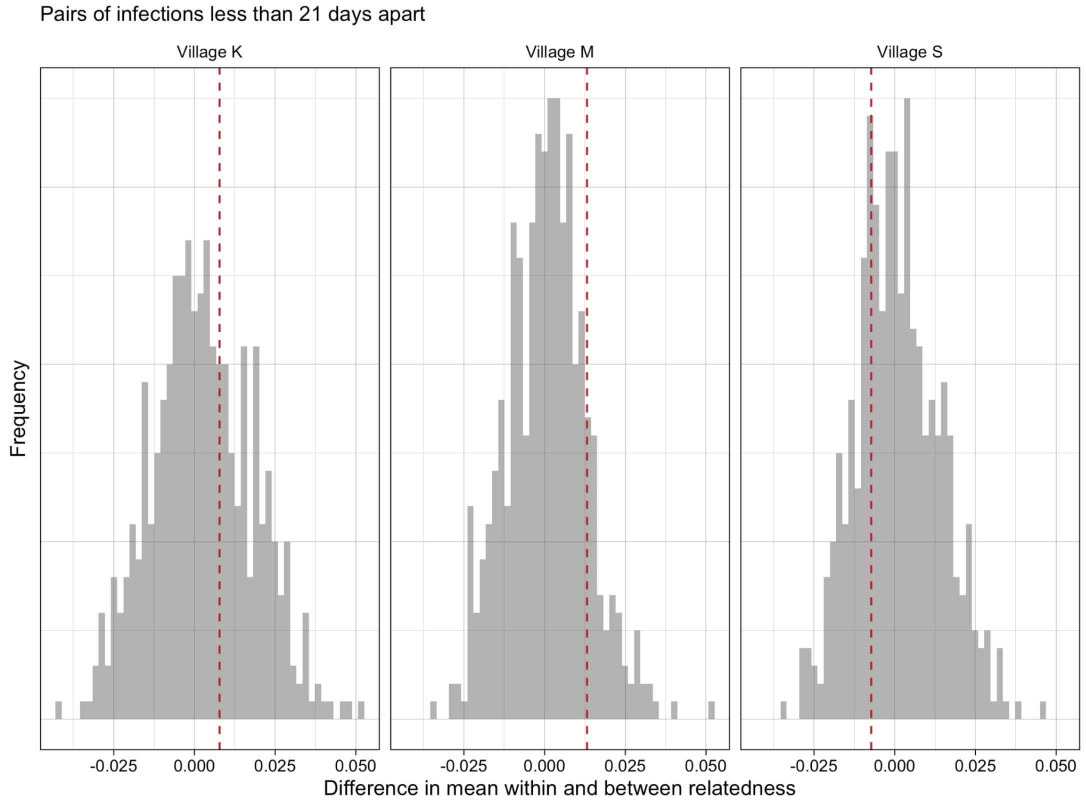

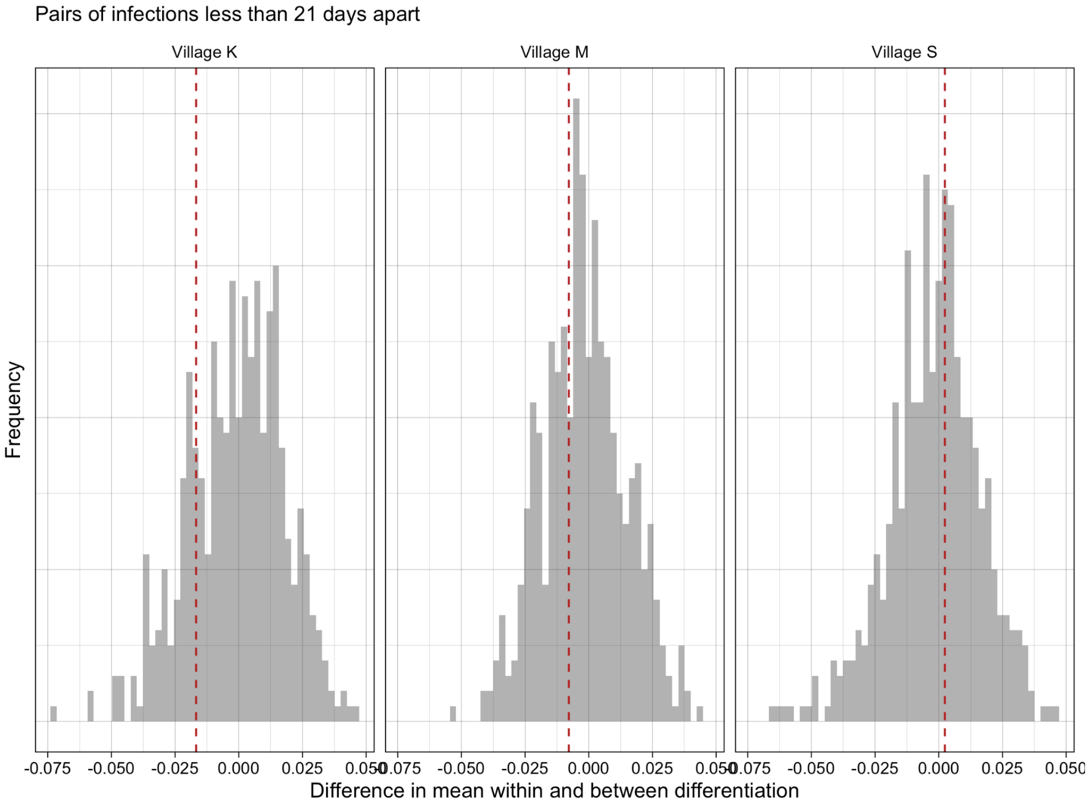

Pairs of infections less than 21 days apart
Village K
Village M
Village S
Frequency
-0.075
-0.050
-0.025
0.000
0.025
0.050
Difference in mean within and between differentiation

Figure S11: Fst and Jost's D measures of population differentiation across the three villages in the cohort study.

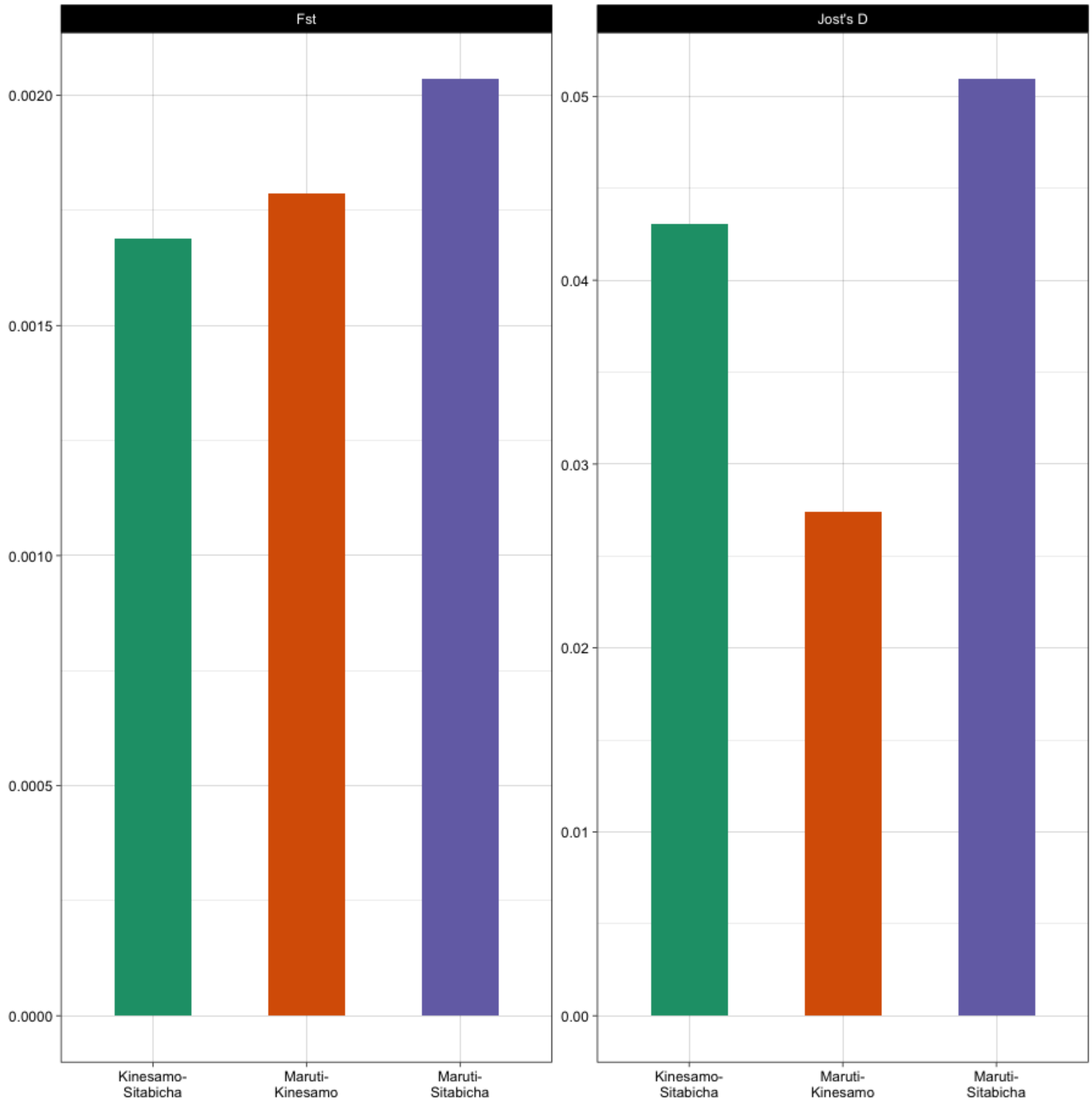

Figure S12: Simulated results of spatial differentiation under various mobility and missingness conditions. (a) Spatial differentiation across multiple simulations and different proportions of the population moving between locations. Values for each location are shown in different colours and the median is shown by the line. (b) Rate that the null hypothesis of no location relatedness structure was rejected at different proportions of random missingness.

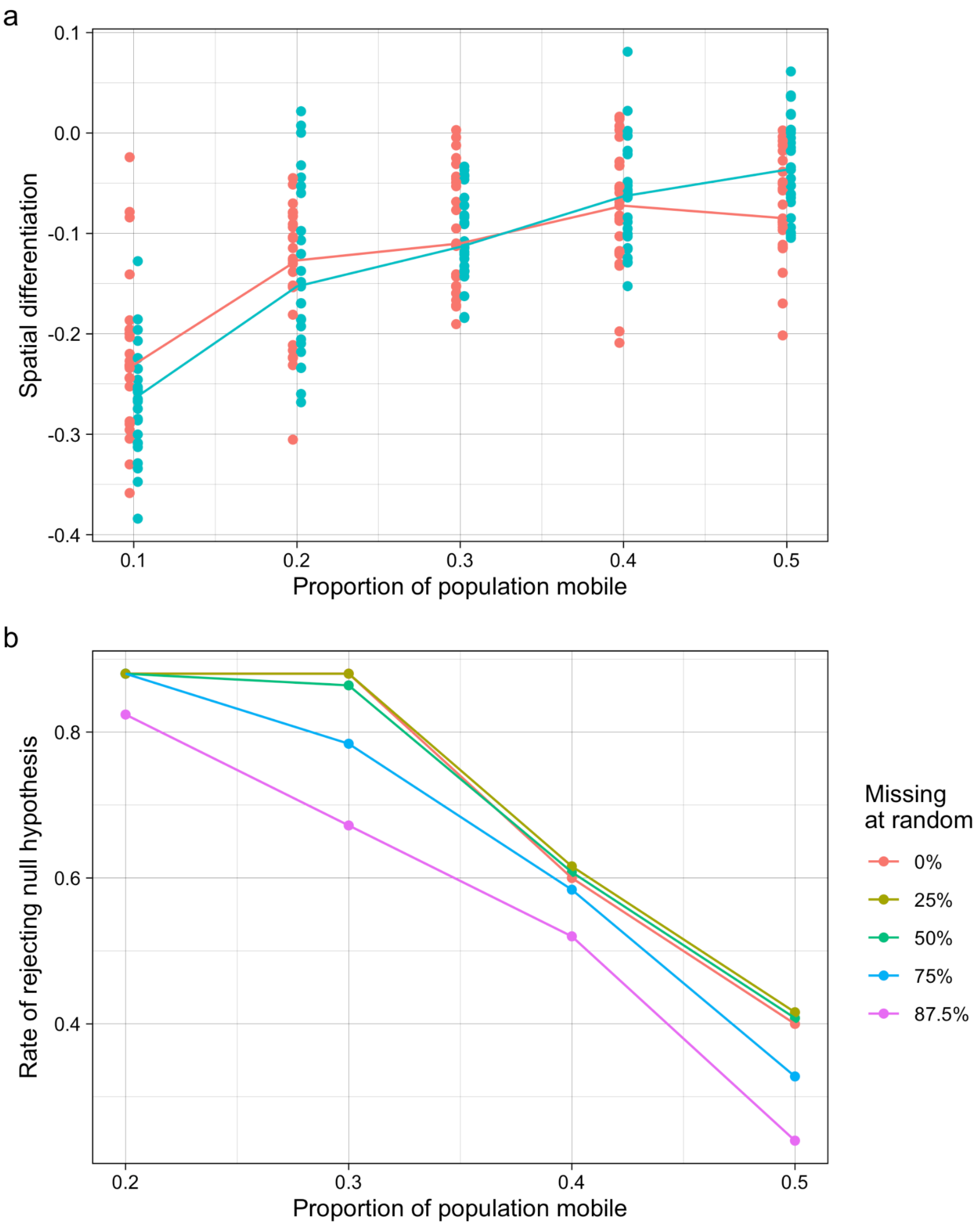

Figure S13: Spatial relatedness with data MCAR. (a) Spatial relatedness at different proportions of asymptomatic and symptomatic infections missing completely at random for location 1. (b) Standard deviation of spatial relatedness across the different sampling repeats in the same simulation at different levels of missingness completely at random.

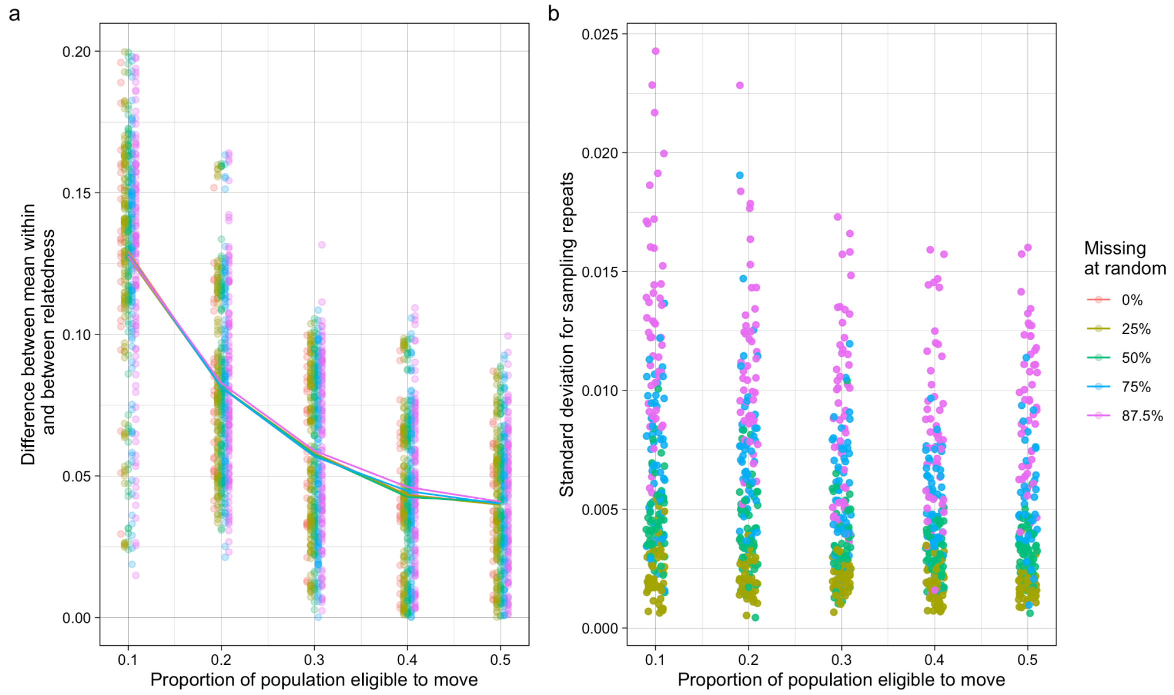

We found that missing only infections with lower-than-average MOI, on the other hand, did bias estimates of genetic similarity across locations upwards. On average, missing 50% of these infections (including both symptomatic and asymptomatic infections) increased the apparent genetic similarity across locations by around 15% on average. Conversely, missing 50% of infections with above average MOI reduced the observed genetic similarity across locations by around 12% (Figure S14).

Figure S14: Average spatial relatedness as proportion of the population moving varied when missing above and below median MOI infections.

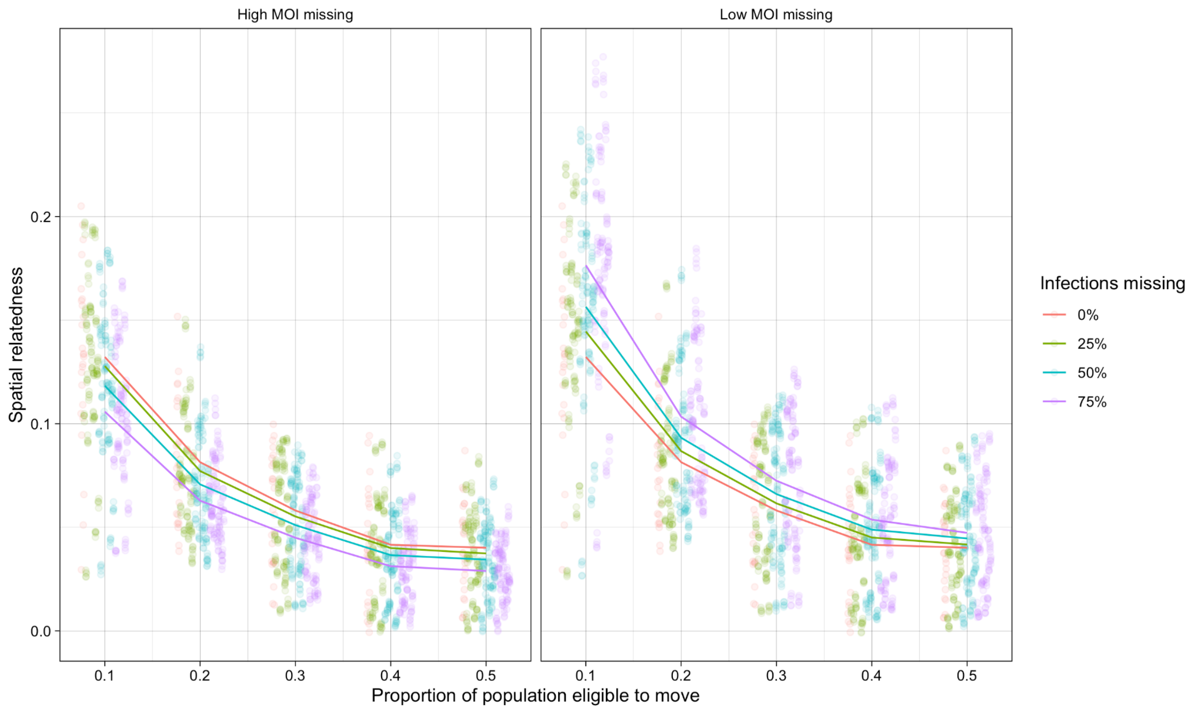

Figure S15: Spatial relatedness with passive case detection. (a) Spatial relatedness and (b) rate of rejecting the null hypothesis when only sampling symptomatic individuals with different levels of missingness.

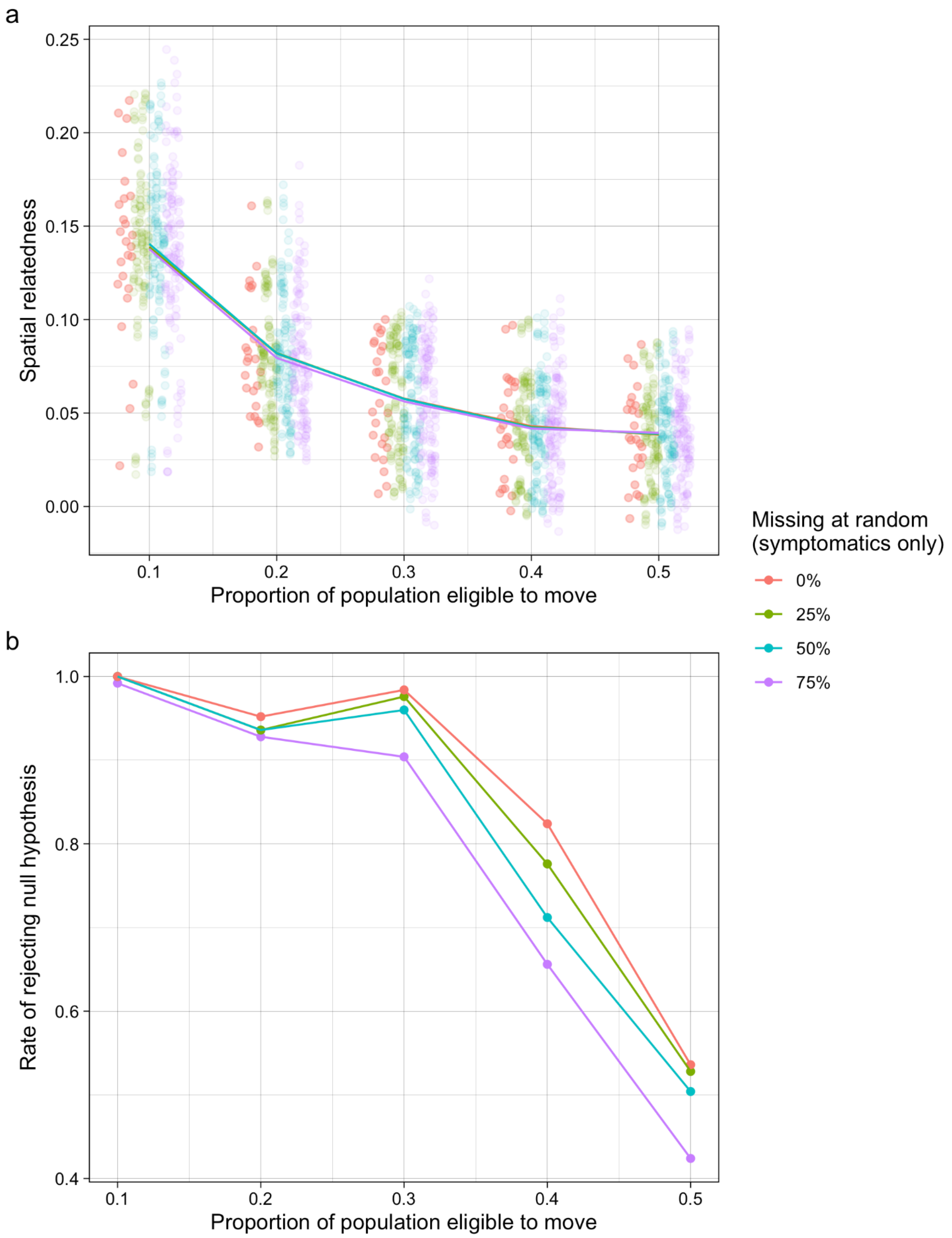

Figure S16: Comparison between Jaccard and relatedness metric, r, in different situations. For each plot the number of haplotypes in the first individual's infection is

fixed. The number of haplotypes in the second individual's infection is then varied along the x-axis and the number of haplotypes in common between the two infections is varied along the y-axis. The colour represents the value of the metric in each situation.

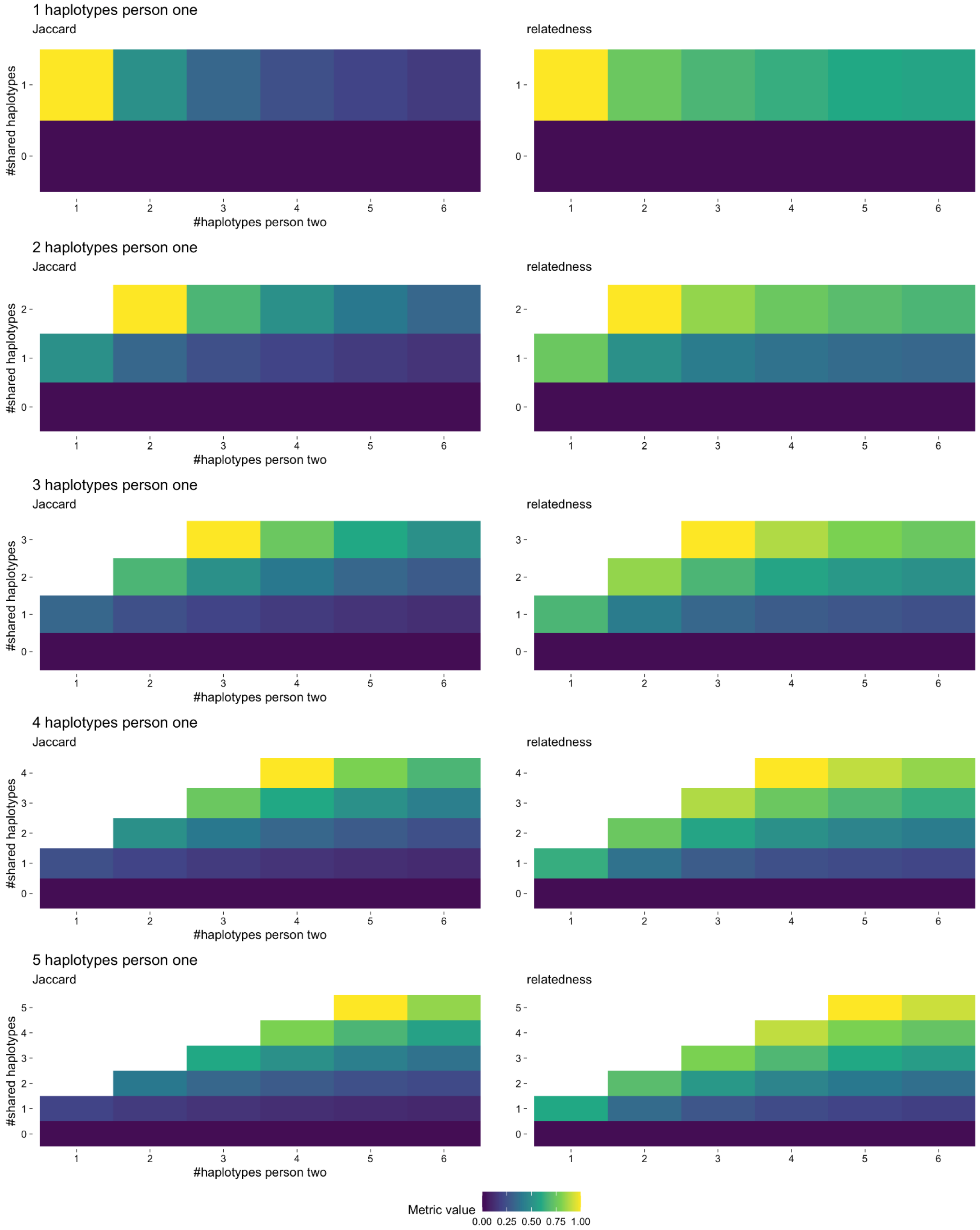

Bretscher, Michael T., Prabin Dahal, Jamie Griffin, Kasia Stepniewska, Quique Bassat, Elisabeth Baudin, Umberto D'Alessandro, et al. 2020. "The Duration of Chemoprophylaxis against Malaria after Treatment with Artesunate-Amodiaquine and Artemether-Lumefantrine and the Effects of pfmdr1 86Y and Pfcrt 76T: A Meta-Analysis of Individual Patient Data." *BMC Medicine* 18 (1): 47.

CDC-Centers for Disease Control, and Prevention. 2009. "CDC - Malaria - About Malaria - Biology," February. https://www.cdc.gov/malaria/about/biology/index.html.

Degefa, Teshome, Delenasaw Yewhalaw, Guofa Zhou, Ming-Chieh Lee, Harrysone Atieli, Andrew K. Githeko, and Guiyun Yan. 2017. "Indoor and Outdoor Malaria Vector Surveillance in Western Kenya: Implications for Better Understanding of Residual Transmission." *Malaria Journal* 16 (1): 443.

Kabbale, Fredrick G., Anne M. Akol, John B. Kaddu, and Ambrose W. Onapa. 2013. "Biting Patterns and Seasonality of Anopheles Gambiae Sensu Lato and Anopheles Funestus Mosquitoes in Kamuli District, Uganda." *Parasites & Vectors* 6 (December): 340.

Mbogo, C. N., E. W. Kabiru, S. K. Muiruri, J. M. Nzovu, J. H. Ouma, J. I. Githure, and J. C. Beier. 1993. "Bloodfeeding Behavior of Anopheles Gambiae S.l. and Anopheles Funestus in Kilifi District, Kenya." *Journal of the American Mosquito Control Association* 9 (2): 225–27.

Ndenga, Bryson, Andrew Githeko, Elizabeth Omukunda, Godwil Munyekenye, Harrysonne Atieli, Peter Wamai, Charles Mbogo, Noboru Minakawa, Goufa Zhou, and Guiyun Yan. 2006. "Population Dynamics of Malaria Vectors in Western Kenya Highlands." *Journal of Medical Entomology* 43 (2): 200–206.

Scott, Thomas W., and Willem Takken. 2012. "Feeding Strategies of Anthropophilic Mosquitoes Result in Increased Risk of Pathogen Transmission." *Trends in Parasitology* 28 (3): 114–21.

Thongsripong, Panpim, James M. Hyman, Durrell D. Kapan, and Shannon N. Bennett. 2021. "Human-Mosquito Contact: A Missing Link in Our Understanding of Mosquito-Borne Disease Transmission Dynamics." *Annals of the Entomological Society of America* 114 (4): 397–414.
